# Impact of chronically alternating light-dark cycles on circadian clock mediated expression of cancer (glioma)-related genes in the brain

**DOI:** 10.7150/ijbs.35520

**Published:** 2019-07-20

**Authors:** Suliman Khan, Yang Liu, Rabeea Siddique, Ghulam Nabi, Mengzhou Xue, Hongwei Hou

**Affiliations:** 1 The Key Laboratory of Aquatic Biodiversity and Conservation of Institute of Hydrobiology, Chinese Academy of Sciences, Wuhan 430072, China.; 2 Key Laboratory of Molecular Biophysics of the Ministry of Education, Wuhan, Hubei 430074, China; 3 University of Chinese Academy of Sciences, Beijing, China.; 4 The Department of Cerebrovascular Diseases, The Second Affiliated Hospital of Zhengzhou University, Zhengzhou, China.; 5 Henan Medical Key Laboratory of Translational Cerebrovascular Diseases, Zhengzhou, China.

**Keywords:** Circadian Rhythm, Clock Genes, Glioma, Oncogenes, Tumor Suppressor Genes.

## Abstract

Disruption of the circadian rhythm is a risk factor for cancer, while glioma is a leading contributor to mortality worldwide. Substantial efforts are being undertaken to decrypt underlying molecular pathways. Our understanding of the mechanisms through which disrupted circadian rhythm induces glioma development and progression is incomplete. We, therefore, examined changes in the expression of glioma-related genes in the mouse brain after chronic jetlag (CJL) exposure. A total of 22 candidate tumor suppressor (n= 14) and oncogenes (n= 8) were identified and analyzed for their interaction with clock genes. Both the control and CJL groups were investigated for the expression of candidate genes in the nucleus accumbens, hippocampus, prefrontal cortex, hypothalamus, and striatum of wild type, *Bmal1^-/-^* and *Cry1/2* double knockout male mice. We found significant variations in the expression of candidate tumor suppressor and oncogenes in the brain tissues after CJL treatment in the wild type, *Bmal1^-/-^* and *Cry1/2* double knockout mice. In response to CJL treatment, some of the genes were regulated in the wild type, *Bmal1^-/-^* and *Cry1/2* similarly*.* However, the expression of some of the genes indicated their association with the functional clock. Overall, our result suggests a link between CJL and gliomas risk at least partially dependent on the circadian clock. However, further studies are needed to investigate the molecular mechanism associated with CJL and gliomas.

## Introduction

Disruption in biological rhythms is linked with various serious psychiatric and brain disorders including mood disorders, depression, anxiety, insomnia, suicidal ideation, Parkinson disease (PD), Alzheimer disease (AD), Prader-Willi syndrome (PWS), Smith-Magenis syndrome (SMS), autism spectrum disorders (ASDs) and attention-deficit hyperactivity disorder (ADHD) 1-3. Similarly, disrupted biological rhythms have been associated with heart diseases, obesity, diabetes, and gastrointestinal dysfunctions 4-6. Shiftwork increases the risk of breast cancer 7 and other malignancies which are caused by a bright light at night through mitigation of pineal hormone melatonin 8. Shiftwork mediated disruption in circadian rhythm can cause cancer by altering regulation of the myelocytomatosis viral oncogene human recombinant (C-myc), alpha protein (Gadd 45a), murine double minute oncogene (Mdm-2) and *p53* encoding genes 8,9. *Per1* and *Per2* are associated with tumor growth 10, whereas altered *Per2* expression dysregulates tumor suppressor genes (cMyc, cyclin D1, cyclin A, Mdm-2, and Gadd45a) and impairs apoptosis by p53 gene 11-13. Altered *PER1*, *PER2,* and *PER3* promote colonic adenoma, colonic cancer, and breast cancer 14,15. The disrupted circadian system induces tumorigenesis in breast and prostate tissues 8, inhibits p53 and enhances the expression of MYC 12,16. Shiftwork suppresses melatonin and related hormones to cause malignancies, cardiovascular, and metabolic disorders 8.

Cancer, the second leading cause of death around the globe that killed 9.6 million people in 2018, has also been linked with the environmentally disrupted circadian *clock*. Although the preclinical data support this link; however, the precise molecular mechanisms underlying the relationship between cancer initiation/progression and *clock* disruption is yet to be understood 7,17,18. The International Agency for Research on Cancer (IARC) listed shift work as a carcinogen (group 2A) for disrupting the biological rhythms 19-21. Several clinical and epidemiological studies revealed a connection between disrupted circadian rhythms and cancer in prostate, breast, and reproductive organs 12,18,20,22. Similarly, several molecular evidence in laboratory studies summarized by Masri and Sassone-Corsi (2018) connect disruption of the circadian molecular machinery with hepatocellular carcinoma, lung cancer, lymphoma, and other tumor types 18. The core clock genes seem essential in tumorigenesis. The SCN, a master circadian *clock,* is an endogenous timekeeping system which further controls many peripheral *clock*s in the peripheral tissues of the body 23,24. Cancer affects the molecular and physiological regulation of brain regions, including nucleus accumbens (NAc), hippocampus, prefrontal cortex (PFC), hypothalamus, and striatum, which leads to the development of psychiatric disorders. Thus, alteration of cancer-related genes in these regions may accelerate the risk of brain cancers 25-29. Although circadian rhythm disruption accelerates tumor progression 8,18 however, there is minimal information available about the circadian rhythm disruption and cancer development in the various tissues of the brain. Therefore, the objectives of our study were to investigate the expression of some vital antitumor and oncogenes in the important brain regions of C57BL/6, *Bmal1^-/-^*, and *Cry1/2* mice after exposure to chronic jetlag like conditions.

## Material and methods

### Animals

Male (C57/BL6) mice were obtained from Model Animal Research Center of Nanjing University. *Bmal1^-/-^* mice is a generous gift from Dr. Lili Chen. *Cry1/2* double knockout mice were generous gifts from Dr. Erquan Zhang. All animals were bred at the SPF animal facility at College of Life Science & Technology, Huazhong University of Science & Technology. These mice were housed in standard cages. The Ambient temperature was 25 ± 1 °C, with food and water available *ad libitum*. Mice remained group housed throughout the experiment. All animal experiments were approved by the Institutional Animal Use and Care Committee at Tongji Medical College, Huazhong University of Science & Technology.

### Light dark cycle conditions

Mice were maintained in light-tight housing cabinets. After acclimatization for one week under 12h light: 12h dark (12L:12D), mice were assigned randomly to LD. The CJL group was exposed to 6h phase advance every two days, whereas the lights on and off times were unchanged for the control group throughout the experimental period. The mice were exposed to jetlag conditions for 30 days. Normal food and water were provided throughout the experimental period.

### Wheel running activity

Mice were randomized into 2 groups; CJL group n(WT=15, *Bmal^-/-^*=12, *Cry1/2*=10) and control group n(WT=15, *Bmal^-/-^*=8, *Cry1/2*=8). The average ages of the mice were 6-8 weeks. All mice were singularly caged and provided with an in-cage running wheel. All mice were given free access to running wheels over a 1-week acclimation period to determine running characteristics of each mouse and to ensure that our randomization was effective in terms of running time and distance. We determined during the acclimatization period that approximately only one percent of wheel activity occurred during the daytime. CJL mice were then exposed to altered light-dark cycles with 6 h phase advance for one month while the control mice were exposed to normal 12L:12D conditions.

### Brain dissection

Tissues were dissected and snap frozen. The brains of mice and the brain regions were dissected following the previously published protocol by Spijker S. (2011) and mouse brain atlas. In brief, after cervical dislocation, the head was removed by using surgical scissors to cut from the posterior side of the ears. A midline incision was made in the skin through scissors. The skin was flipped over the eyes to free the skull. A small incision was made on the top of the skull with care to avoid damaging the brain. Then a cut was made through the most anterior part of the skull or frontal bone. Hence the brain was removed. Hippocampus was removed by placing the brain with the ventral side facing the metal plate. Closed small curved forceps were placed between the cerebral halves, and the brain was held with largely curved forceps. The forceps were gently opened to open the cortical halves. This step was repeated until the complete opening of the regions. After opening around 60% along the midline, the left cortex was opened from the hippocampus by repeatedly opening the forceps in closed position 30-40° counterclockwise. Small forceps were used to separate the hippocampus from the fornix. Hence, two halves of the hippocampus were separated. Medial prefrontal cortex and striatum extracted through coronal sections approximately 1.0 mm using a sharp and clean blade. Anterior commissure became visible after cutting the olfactory bulb. The first section contains motor cortex, while the subsequent section contains the anterior corpus callosum with a darker area in the prefrontal cortex (PFC). Next ventral-dorsal striata were separated. Hypothalamus was located via optic chiasm which was away from the anterior portion of the hypothalamus, followed by dissection of the mammillary nuclei from the posterior of the hypothalamus. For raphe nuclei, the two main sections (approximately 1.5-2.0 mm) were cut off from the brain and then a comparatively thinner section approximately, 1.0 mm was cut off. The midbrain was exposed by cutting off the upper gray matter regions, and raphe nucleus was exposed and scooped out. Nucleus accumbens (NAc) was dissected by following the previously published protocol with necessary modifications 30. In brief, the brain was trimmed to expose an angled parasagittal surface and cut sharp blade to yield approximately 1 mm thick brain slice that contained most of the ipsilateral fornix and the central portion of the NAc. The NAc was dissected out with care to avoid cutting out the other brain parts. All the brain regions were immediately transferred to liquid nitrogen soon after the dissection and stored in -80°C before further use. The brain slices were measured using vibratome. The fresh brain was used to extract higher quality RNA quality. For all dissection experiments, mice were transported to the experimental room and acclimatized for one hour. The brain dissection was performed at ZT^2^-^3^. To avoid ambiguity and to provide equal opportunity, all the mice were dissected in three independent experiments by repeating the order of dissection according to mice type. Each experimental group consisted of 7 mice, while 2-3 mice/ group were dissected each time experiment. n(WT/control=7, WT/CJL=7; *Bmal1^-/-^*/control=7, *Bmal1^-/-^*/CJL=7; *Cry1/2* double knockout-control=7, *Cry1/2* double knockout-CJL=7).

### RNA extraction

Total RNA was extracted using TRIzol™ reagent (ThermoFisher SCIENTIFIC, China) following the standard protocol provided by the manufacturer. 1 mL of TRIzol™ reagent per 50-100 mg of tissue was added, and samples were homogenized using a hand-held homogenizer. Samples having high-fat contents were centrifuged for 5 min at 12,000 × g and 4°C. The supernatant was transferred to a new tube. Samples were incubated for 5 min to permit complete dissociation of the nucleoprotein complex. 0.2 mL of chloroform (trichloromethane, CHCl_3_) was then added per 1 mL of TRIzol™ Reagent. After incubation for 2-3 min, the samples were centrifuged for 15 min at 12,000 × g and 4°C. The mixture was separated into a lower red phenol-chloroform, interphase, and a colorless upper aqueous phase. The aqueous phase containing the RNA was transferred into a new tube. An equal volume of ethanol was then added to the aqueous phase and incubated for 10 min. The samples were subsequently centrifuged for 10 min at 12,000 × g in 4°C. The supernatant was discarded with a micropipette. The pellet was washed with 1 mL of 75% ethanol per 1 mL of TRIzol™ Reagent used by vortexing briefly and then centrifuge for 5 min at 7500 × g in 4°C. After discarding the supernatant with a micropipette, the RNA pellet was air dried for 15-20 min. The pellet was resuspended in 20-50 μL of RNase-free water according to the weight of tissue used for RNA extraction. The tubes were heated at 55-60°C for 10 minutes. Then RQ1 buffer (RNAse free DNAse buffer) and RQ1 RNase free DNAse enzyme (SolarBio Science and Technology, Beijing, China) was added into the tubes and heated at 37°C for 30 min. Next, stop enzyme was added and heated at 65°C for 10 min to stop the reaction. The RNA concentration was determined using spectrophotometer by the following formula


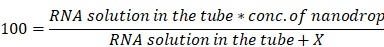


### Primers design

Primers were designed using PRIMER BLAST, PRIMER-3, and PRIMER-5 (Table 1). The average melting temperature was selected as 60°C, and PCR product size was 70-200 bp. Standard primer size selected was 20bp, whereas average *GC* content (%) was 50%. All the primers were purchased from AuGCT DNA-SYN Biotechnology, China.

### Quantitative PCR (qPCR)

cDNA was synthesized using high capacity RNA to cDNA kit (Transgen, Biotech, China). qPCR was performed using Trans Master Mix (Transgen, Biotech, China) and an applied biosystems 96 well thermal cycler (ThermoFisher SCIENTIFIC). Primer sequences are listed in Table S1. Master mix was prepared according to the manufacturer's protocol with certain modifications according to the requirements. For each reaction, 10 μL qPCR mix (2X), 0.4 µL forward primer (1nmol), 0.4 µL of reverse primer (1nmol) and 0.4 μL reference dye were used per reaction. 1 µL or 1.4 µL of cDNA or RNA (200 or 100 ng/µL) was added to each reaction. The reactions were then run using the following condition: initiation temperature at 94 °C for 2-10 min followed by 40 cycles with denaturation temperature set at 94 °C for 15 seconds and annealing temperature at 60 °C for 1 min. After completion of the reaction, fold changes of expression of genes of interest were normalized to GAPDH endogenous reference gene and then normalized to control samples, and calculated using the ΔΔCt method 31.

### Data resources and cancer-associated genes selection

We used the Candidate Cancer Gene Database (CCGD) to download available cancer-associated genes (http://ccgd-starrlab.oit.umn.edu/about.php) 32. A total of 10523 genes were found in the database. For confirmation, these genes were aligned to a total of 2164 genes downloaded from cancer genetics database (http://cancer-genetics.org/genes_download.txt). The aligned 1213 genes were further aligned with 698 protein-coding oncogenes downloaded from oncogene database (http://www.ongene.bioinfo-minzhao.org/) considering the genes reported in human 33. For the sake of removing ambiguity, we further aligned the selected 272 genes with a total of 713 cancer genes downloaded from the Network of Cancer Gene Database (NCG6.0) (https://www.facs.org/quality-programs/cancer/ncdb) 34. At this stage, we finalized 185 genes which were mutually present in these datasets. Genes lists were downloaded from Human Brain Transcriptome 35,36 through online browsing (http://hbatlas.org/pages/hbtd) and aligned to obtain to the region-specific genes. Finally, 133 genes were identified among all the datasets. For antitumor genes, we first downloaded Tumor Suppressor Gene Database (TSGD) via an available online link (https://bioinfo.uth.edu/TSGene/). A total of 1217 genes were downloaded 37. These genes were aligned with genes downloaded from HBT to select genes expression in the brain. To find out the genes under the regulation of the circadian system, we further aligned these genes with the genes sets of Circadian Gene Database (CGDB) 38 (http://cgdb.biocuckoo.org/links.php) and Circadian Expression profile database (http://circadb.hogeneschlab.org/human) 39. Finally, a total of 68 antitumor genes were identified mutually in related databases. In the case of HBT, the genes in pituitary and nerve tibial were combined and aligned with a regional module of HBT, and successful candidates were further processed.

### Sequence extraction and phylogenetic analysis

To find out sequences for the selected genes, we used the *Genebank*/*FASTA* sequence (NCBI) database (https://www.ncbi.nlm.nih.gov/nuccore). We used the browse function NCBI (Gene) to search for the sequences of a total of 201 genes through their gene IDs. For the sake of simplicity, we selected only one variant of each gene for phylogenetic analysis. All the pre-selected genes were subjected to *MEGA 6.0.6* software for phylogenetic analysis 40, and all those sequences that clad at the same level were taken as relevant genes. The phylogenetic tree was constructed using sequence alignment and phylogeny options. Duplicates and poorly aligned sequences were removed before building a tree.

### Cancer-associated genes annotations and bioinformatics analysis

The selected genes were subjected to functional annotation analysis using DAVID Bioinformatics Resources 6.8 (https://david.ncifcrf.gov/summary.jsp) for finding the associated KEGG pathways 41. Top ten pathways were selected, and topmost genes were compared with the similar clads of the phylogenetic tree. The gene symbols were converted to ensemble IDs using BioDBnet (https://biodbnet-abcc.ncifcrf.gov/db/dbOrthoRes.php) before their KEGG analysis. To find the interactions of molecular pathways, the genes were subjected to CPDB database (http://cpdb.molgen.mpg.de/). Induced network modules and enrichment analyses were carried out to determine the specific interactions and genetic level, post-genetic level, and biochemical level 42,43. Based on the KEGG analysis and phylogenetic tree representation, 33 genes were selected.

### Tissue level expression and pattern analysis

The selected 22 representative cancer-related genes were further analyzed for their specific expression in brain and other tissues. Mean expression values of genes in the human brain were downloaded from HBT, whereas the tissue-specific expression values were downloaded using the NCBI gene browser using *mus musculus* as the target species (https://www.ncbi.nlm.nih.gov/gene). The data were subjected to the “expression-based pretty heat map” tool of imageGP, an online tool for converting numerical data into heatmaps using log2 through Pearson distance matrix method with default remaining conditions (http://www.ehbio.com/ImageGP/index.php/Home/-Index/PHeatmap.html). Overall, genes identified through different databases were subjected to Venn diagrams for their proper representation using Venny 2.1 (http://bioinfogp.cnb.csic.es/-tools/venny). Furthermore, SCNseq online database was used to determine the *ZT* timepoint for maximum and minimum expression of selected genes in SCN (http://www.wgpembroke.com/shiny/SCNseq/).

### Statistical analysis

All results presented were analyzed using Excel (Microsoft Software, 365) and GraphPad Prism 7 (GraphPad Software, La Jolla, California, USA). Student's T-test, one-way and two-way ANOVA have performed accordingly. *P* value <0.05 was considered significant.

## Results

### Identification and selection of candidate genes

**Figure 1:**
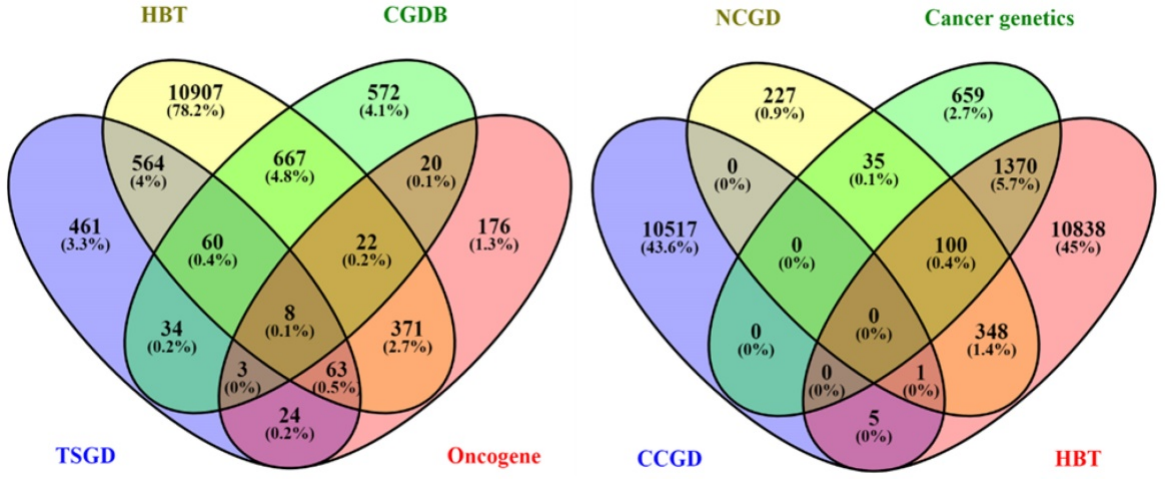
Distribution of genes downloaded using different databases. Venn diagram shows the number of downloaded genes from all selected databases (HBT, CGDB, TSGD, Oncogene, NCGD, Cancer genetics, CCGD, and HBT) and the distribution of genes across the databases. The matched genes among all databases were selected for further process.

Oncogenes and tumor suppressor genes were selected in the peer-reviewed literature using relevant databases, including CCGD, cancer genetics database, OGDB, NCG6.0, HBT, TSGD, CGDB, and CircaDB (Figure 1). The schematic overview of the experimental process has been shown in Figure 1.

**Figure 2:**
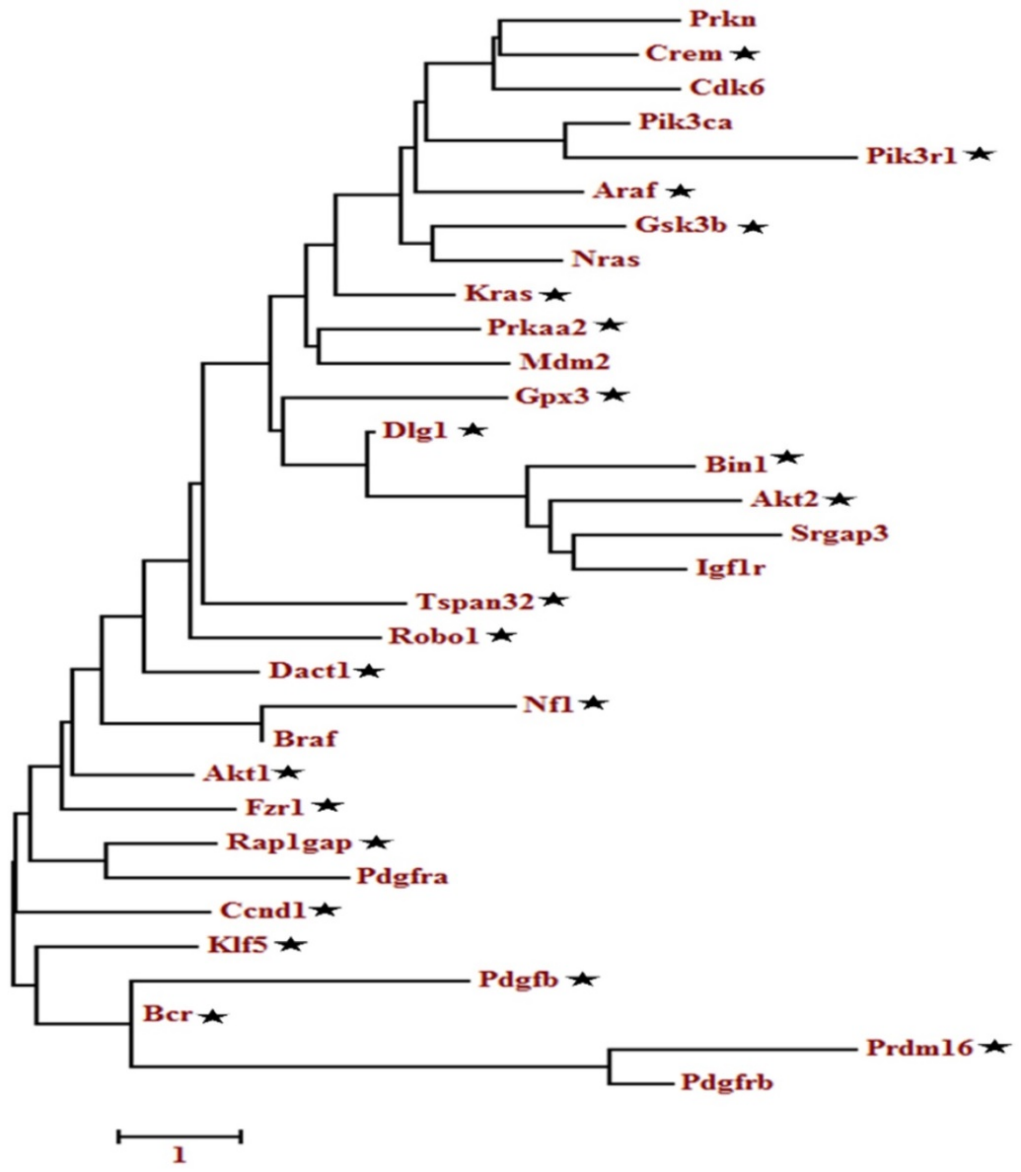
Molecular phylogenetic analysis of the selected tumor suppressor and oncogenes genes. The evolutionary history was inferred by using the Maximum likelihood method based on the Tamura-Nei model. The tree with the highest log likelihood is shown. Initial tree(s) for the heuristic search were obtained automatically by applying Neighbor-Join and BioNJ algorithms to a matrix of pairwise distances estimated using the Maximum Composite Likelihood (MCL) approach and then selecting the topology with superior log-likelihood value. A discrete Gamma distribution was used to model evolutionary rate differences among sites. The tree is drawn to scale, with branch lengths measured in the number of substitutions per site. The analysis involved, 32 nucleotide sequences. Codon positions included were 1st+2nd+3rd+Noncoding. All positions containing gaps and missing data were eliminated. Evolutionary analyses were conducted in MEGA6.

A total of 133 oncogenes and 68 tumor suppressor genes were found in all the databases (Table S4). KEGG pathway analysis showed that among the 201 unigenes (both oncogenes and tumor suppressor genes), 33 genes were implicated in the glioma pathway (Figure S1). The pathway-based analysis further revealed that four tumor suppressor genes (*Per2, Prkaa2, Npas2, Arntl)* were associated with circadian rhythm pathway. The phylogenetic analysis of the selected 201 genes (tumor suppressor and oncogenes) as depicted in Table S4, showed a relationship between oncogenes and tumor suppressor genes (Figure S2-S4). Among the closely related genes, 32 genes found appeared repeatedly in topmost cancer pathways (especially glioma) during KEGG analysis. These identified genes were further analyzed using phylogenetic analysis to determine the candidate genes (Figure 2). Finally, 22 candidate genes were selected based on the criteria that at least one representative gene was selected per clade. The ZT timepoints for maximum and minimum expression levels were determined using the online database SCNseq (Table 2).

The evolutionary history was inferred by using the Maximum likelihood method based on the Tamura-Nei model. The tree with the highest log likelihood is shown. Initial tree(s) for the heuristic search were obtained automatically by applying Neighbor-Join and BioNJ algorithms to a matrix of pairwise distances estimated using the Maximum Composite Likelihood (MCL) approach and then selecting the topology with superior log-likelihood value. A discrete Gamma distribution was used to model evolutionary rate differences among sites. The tree is drawn to scale, with branch lengths measured in the number of substitutions per site. The analysis involved, 32 nucleotide sequences. Codon positions included were 1st+2nd+3rd+Noncoding. All positions containing gaps and missing data were eliminated. Evolutionary analyses were conducted in MEGA6.

### Candidate cancer-related genes are predicted to interact with clock genes

**Figure 3:**
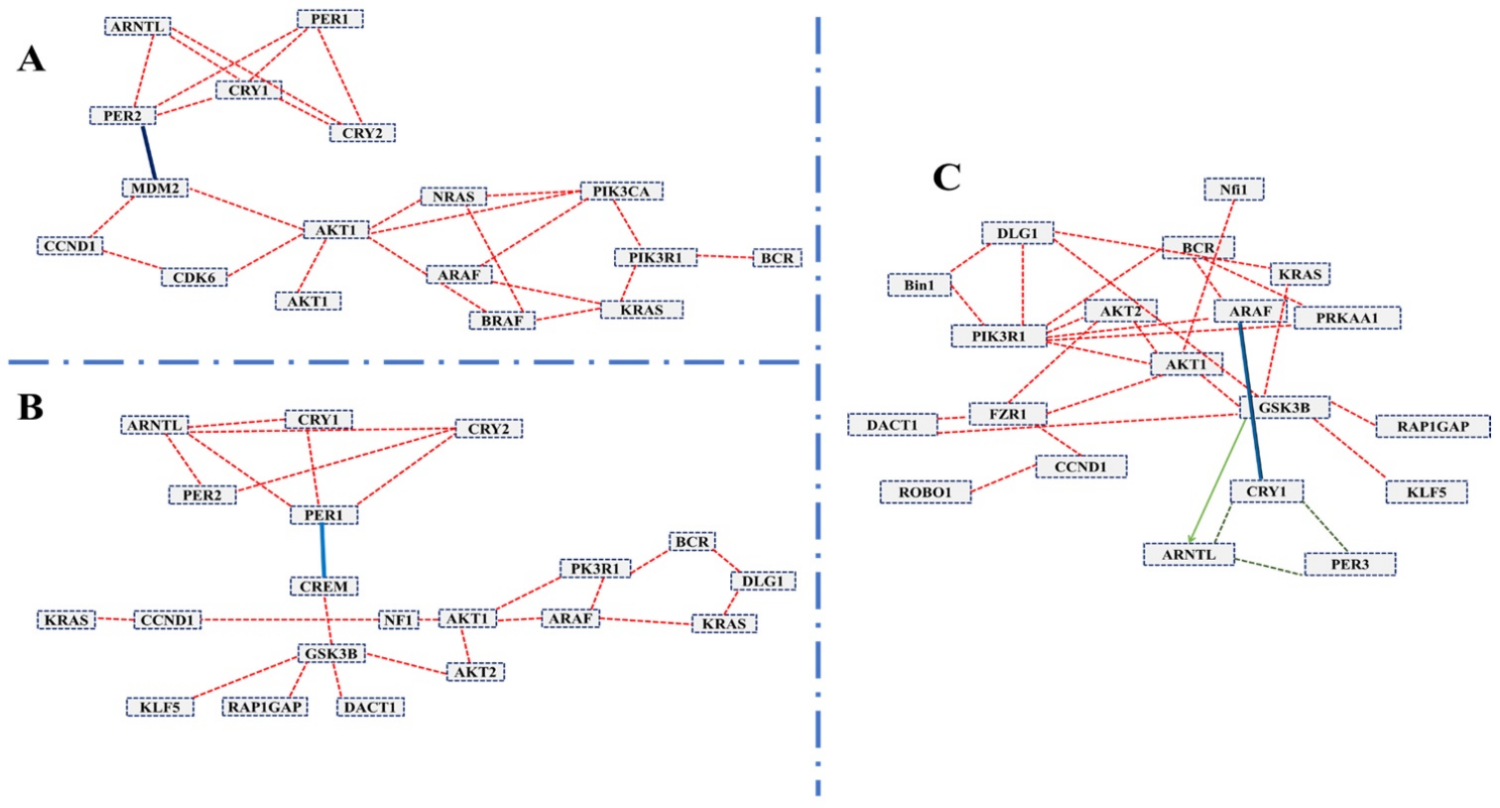
The induced network was analyzed using the CPDB online database by considering the gene interaction, protein interaction, and biochemical interaction without intermediate links. The interactions between cancer-related genes and clock genes were determined. (A)- MDM2 is associated with PER2 in glioma (B)- CREM is associated with PER1 in myeloid leukemia and (C)-GSK3 is associated with ARNTL in prostate cancer. The cancer genes interacted with clock genes have been indicated with a blue line and green arrow.

**Figure 4:**
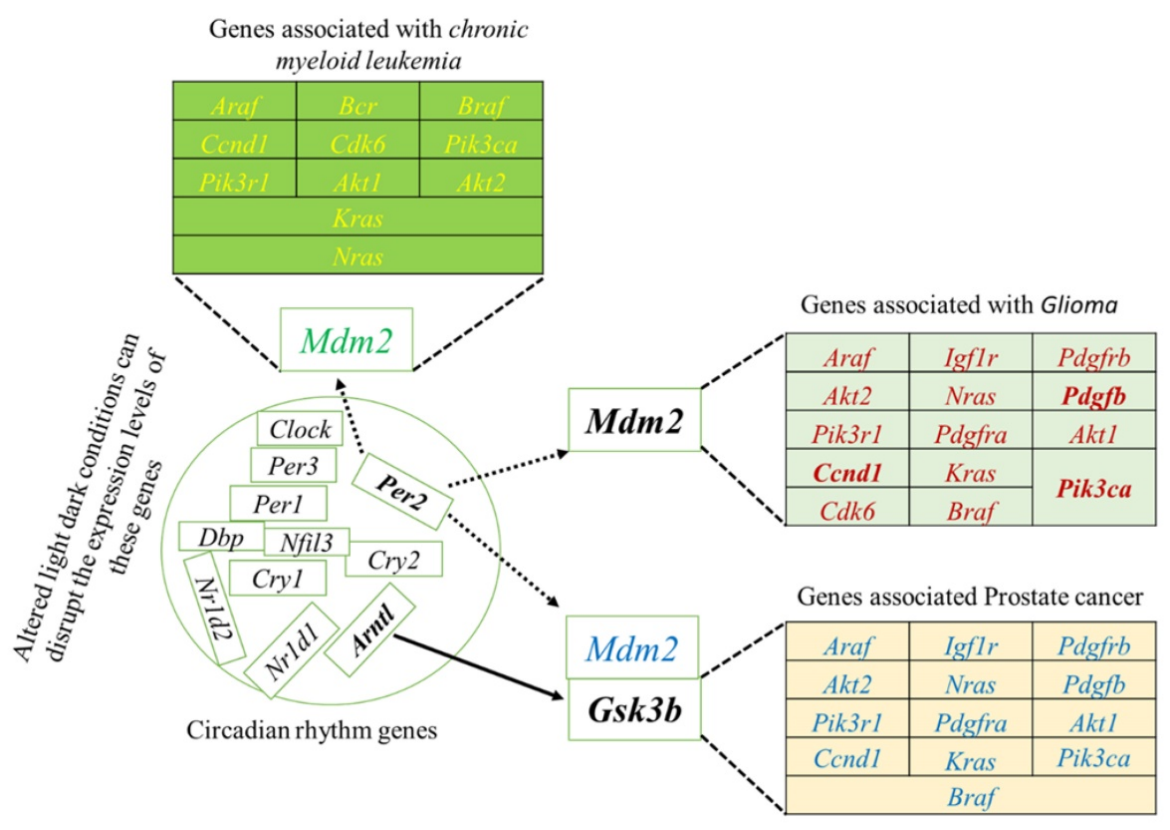
Schematic network based on CPDB analysis. This figure shows the putative schematic diagram for the interaction of clock genes with cancer-associated genes. *Mdm2* and *Gsk3b* were detected to provide the connection between cancer-related genes and clock genes.

To systematically study the complex biological function and associated pathways of the genes, we mapped the assembled 22 (candidate) unigenes against the KEGG analysis tool using DAVID database and found 15, 15 and 12genes associated with glioma, prostate cancer, and chronic myeloid leukemia, respectively (Table S2). These genes were associated with 83 KEGG pathways (Table S2). We further investigated the interaction between clock genes and selected candidate genes, at the gene level, protein level, and biochemical level using the CPDB online database. We found that the clock genes (*Arntl, Per1, Per2)* interact with the candidate tumor suppressor and oncogenes *via Gsk3b*, *Crem,* and *Mdm2* (Figure 3). *Mdm2* and* Crem* are associated with glioma, leukemia, prostate cancer, chronic myeloid leukemia, and several other cancer pathways, whereas, *Gsk3b* is an antitumor gene associated with prostate cancer (Table S3). A putative schematic network was generated to summarize and characterize the selected functional genes in cancer pathways (Figure 3 A-C, Figure 4).

### The selected candidate genes express in various organs

**Figure 5:**
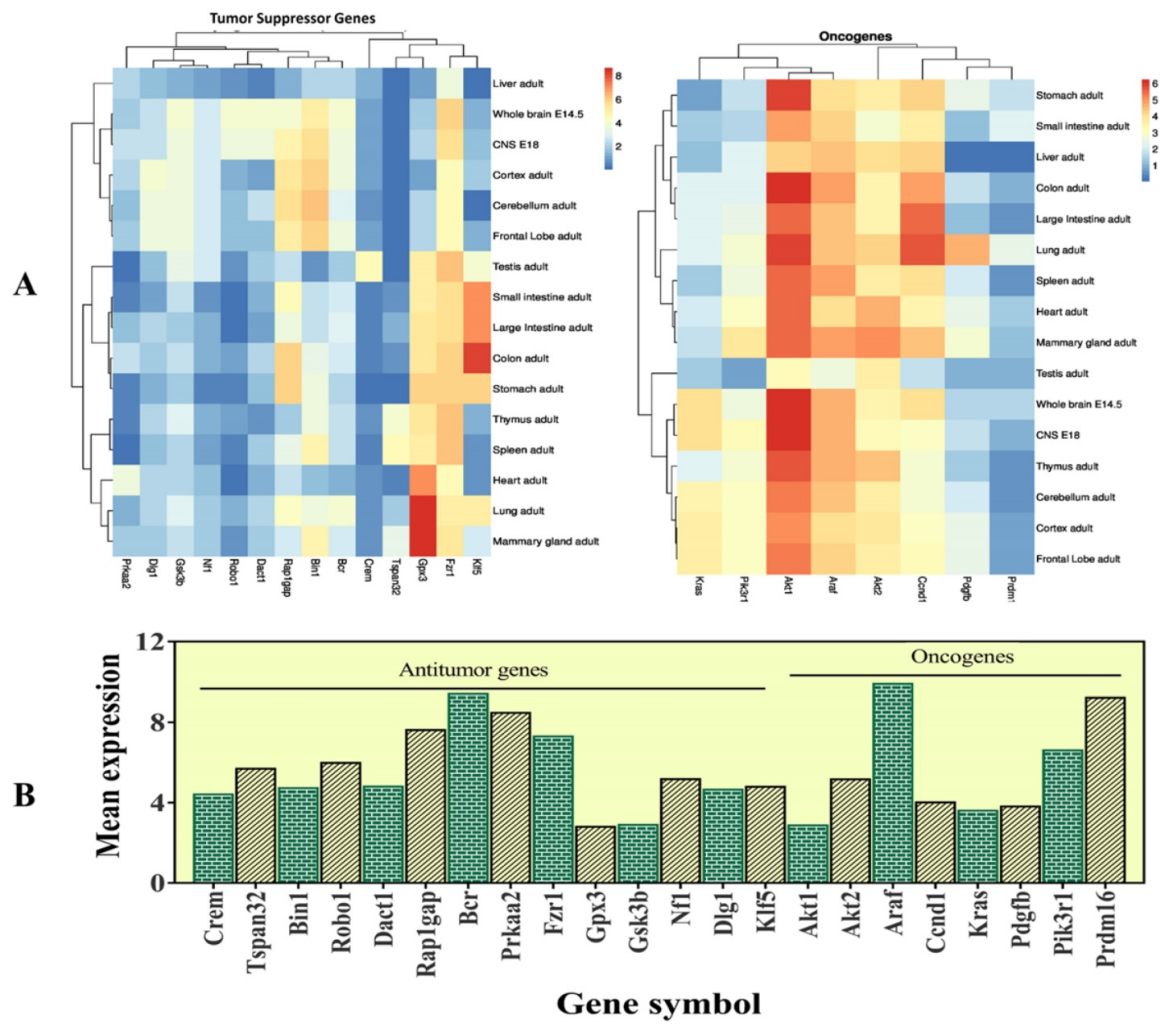
This figure represents the expression levels of the selected genes. A- heatmap of the selected genes to determine their RPKM values in different organs of the body and different brain regions. B- mean expression levels in the brain.

The selected 22 candidate genes (Table 2) were searched using the NCBI browser for their expression pattern in different organs and brain areas. The HBT downloaded data was searched for mean expression levels of genes in the human brain. The expression patterns showed that 22 selected genes related to cancer and tumor suppression were expressed in the central nervous system (CNS), whole brain, cortex, cerebellum, liver, intestine, and frontal lobe. Most of them were found with low expression levels in the entire brain. However, *Crem* and* Tspan32* showed the lowest and *Fzr1,* and *Bin1* showed the highest expression levels. Overall, the highest expression levels were observed for *Gpx3* (mammary glands and lungs), *Klf5* (colon), and *Gsk3b* (brain) (Figure 5). Among the selected genes, 15 (*Araf, Braf, Kras, Ccnd1, Cdk6, Igf1r, Nras, Pik3ca, Pik3r1, Pdgfra, Pdgfrb, Pdgfb, Akt1, Akt2,* and* Mdm2*) were related to glioma and leukemia (Figure 4).

### Exposure to CJL conditions shifted the phase of wheel-running activity

**Figure 6:**
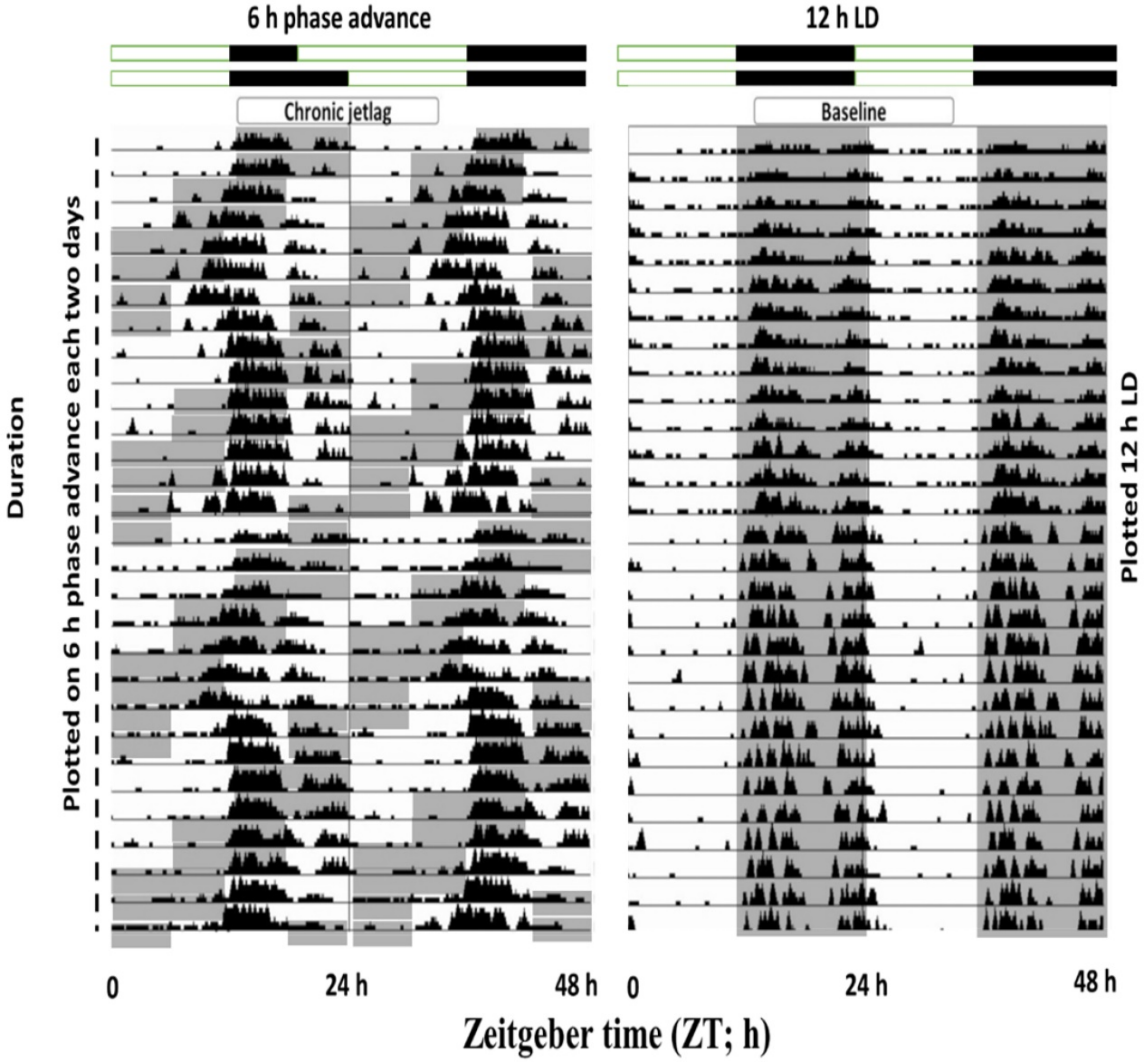
Double-plotted actograms from baseline and CJL mice groups. Baseline mice maintained under 12-hour light/dark conditions (Right side). CJL group was exposed to 6 h phase advance each 2-3 days. Gray-shaded areas of actograms delineate lights off (dark).

To understand the effects of shiftwork on mice physiological behavior, we first employed an established CJL model by exposing mice to a 6 h phase shift every 2-3 days (figure 6). Wheel running activities were recorded which confirmed that activity was shifted by 6 h phase advance of the LD cycle. Those mice showed abnormal wheel run activity were excluded from the experimental process.

### CJL altered the brain region-specific expression levels of candidate genes in mice with intact circadian clock

**Figure 7:**
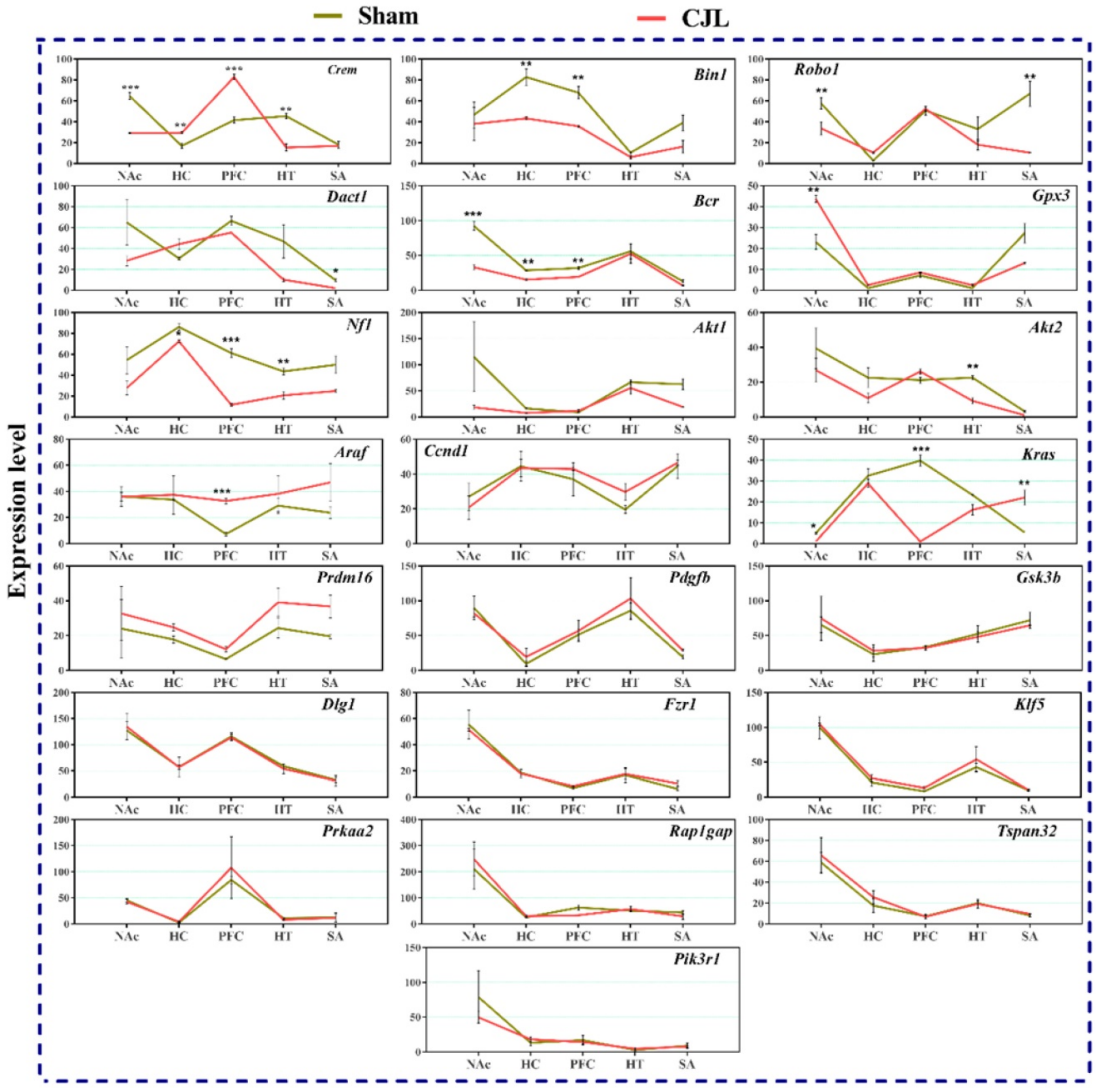
CJL altered mRNA levels of genes associated with cancer in wild type mice. This figure shows mRNA levels in brain regions (NAc, hippocampus, PFC, hypothalamus, and striatum) extracts from sham and CJL treated animals (n = 7 per group), as assayed by three independent qPCR assays. Expression levels in CJL treated mice were normalized to selected expression levels in baseline mice. Results are expressed as mean ± SEM. *P < 0.05, **P < 0.01, ***P < 0.001, two-way ANOVA.

We investigated the mRNA levels in C57BL/6 male mice, exposed to CJL for a period. We found that *Crem* was downregulated in NAc and hypothalamus and upregulated in PFC and hippocampus compared to control. In the CJL group, *Bin1* was downregulated in the hippocampus and PFC. Both the *Robo1* and *Dact1* were downregulated in the striatum. However, *Robo1* was upregulated in the hippocampus. Our results revealed that the *Bcr* was downregulated in NAc, hypothalamus, and PFC. Similarly, *Nf1* was downregulated in the hippocampus, PFC and hypothalamus. In contrast, *Gpx3* was upregulated in NAc. *Akt2* was downregulated in hypothalamus whereas *Akt1* remained the same throughout the brain regions after CJL in comparison with control. Furthermore, *Kras* was upregulated in striatum and NAc. The levels of *Araf*, *Prdm16*, *Ccnd1*, *Pdgfb*, *Pik3r1*, *Gsk3b*, *Dlg1*, *Fzr1*, *Klf5*, *Prkaa2*, *Rap1gap*, and *Tspan32* remained unaltered in all the brain regions in response to CJL treatment compared with control (Figure 7).

### CJL altered the brain region-specific expression levels of candidate genes in *cry1/2* knockout mice

**Figure 8:**
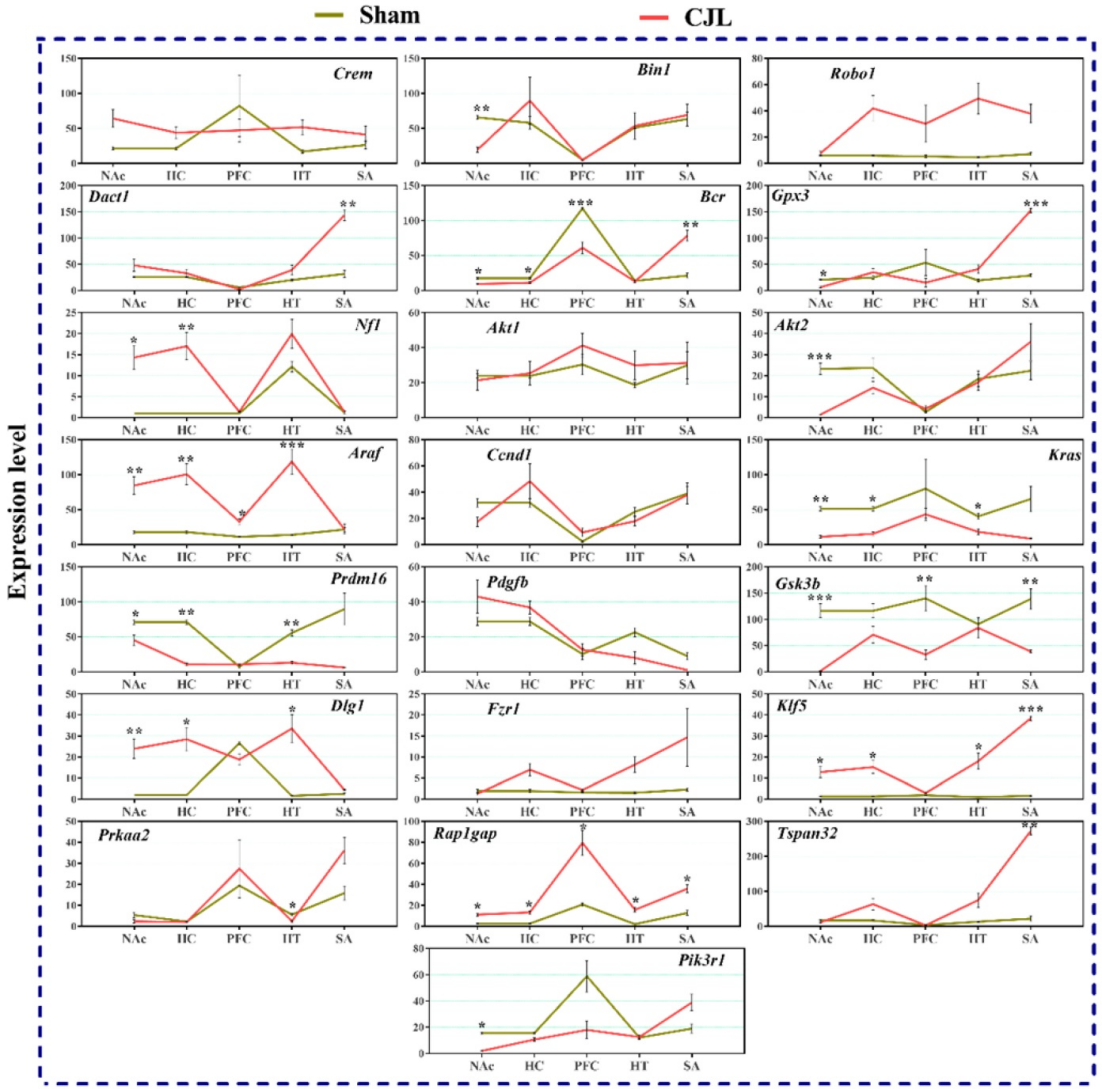
CJL altered mRNA levels of genes associated with cancer in *Cry1/2* mice. This figure shows mRNA levels in brain regions (NAc, hippocampus, PFC, hypothalamus, and striatum) extracts from sham and CJL treated animals (n = 7 per group), as assayed by three independent qPCR assays. Expression levels in CJL treated mice were normalized to selected expression levels in baseline mice. Results are expressed as mean ± SEM. *P < 0.05, **P < 0.01, ***P < 0.001, two-way ANOVA.

*Cry1/2* double knockout mice exposed to CJL showed significant variations in the expression of selected genes in the different brain areas, as shown in figure 8. In summary, *Dact1* and *Tspan32* were upregulated in the striatum. In NAc, several genes (*Pik3r1, Akt2, Gpx3, Kras, Gsk3b*, and *Bin1*) were downregulated. The genes (*Araf*1, *Dlg1*, *Klf5*, and *Rap1gap*) showed upregulations while *Bcr, Kras*, and *Gsk3b* were downregulated in three or more brain regions after CJL. However, the genes (*Crem, Robo1, Akt1, Ccnd1, Pdgfb,* and *Fzr1*) showed no variations in any of the brain region (Figure 7).

### CJL altered the brain region-specific expression levels of candidate genes in Bmal1^-/-^ knockout mice

**Figure 9:**
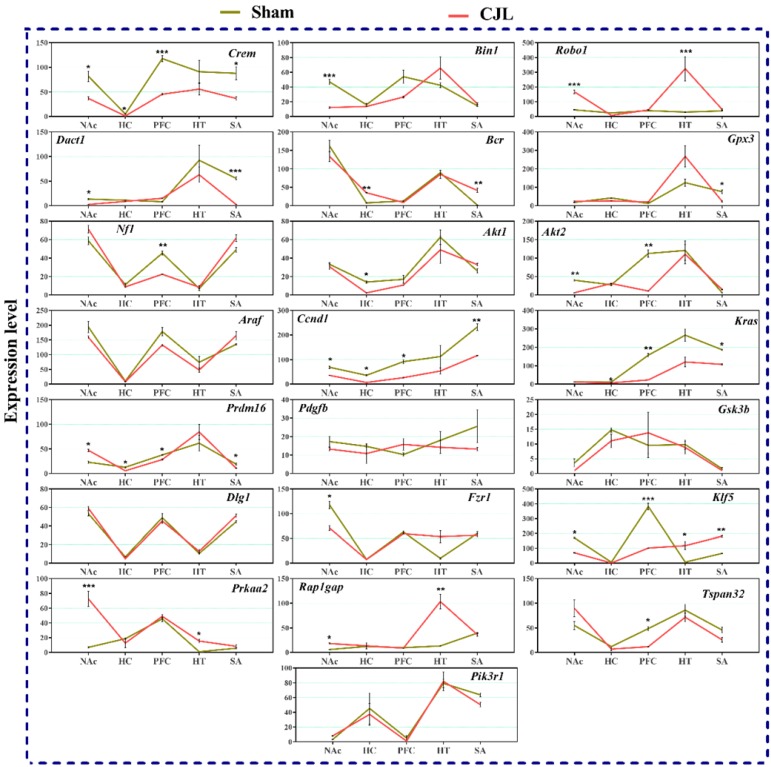
CJL altered mRNA levels of genes associated with cancer in *Bmal^-/-^* mice. This figure shows mRNA levels in brain regions (NAc, hippocampus, PFC, hypothalamus, and striatum) extracts from sham and CJL treated animals (n = 7 per group), as assayed by three independent qPCR assays. Expression levels in CJL treated mice were normalized to selected expression levels in baseline mice. Results are expressed as mean ± SEM. *P < 0.05, **P < 0.01, ***P < 0.001, two-way ANOVA.

Figure 9 indicate variations in mRNA levels in the brain regions of *Bmal1^-/-^* knockout mice exposed to CJL. In short, five of the genes (*Ccnd1, Kras, Klf5, Crem,* and* Prdm16*) showed downregulations in three or more brain regions. Overall, several genes (*Bin1, Dact1, Gpx3, Nf1, Akt1, Akt2, Fzr1,* and* Tspan32*) showed downregulation, and several genes (*Robo 1, Bcr, Klf5, Prkaa2*, and *Rap1gap*) showed upregulation in one or more brain region after CJL (Figure 9).

### Clock genes (*Bmal1*, *Cry1/2*) regulate the expression of cancer-related genes in the brain

**Figure 10:**
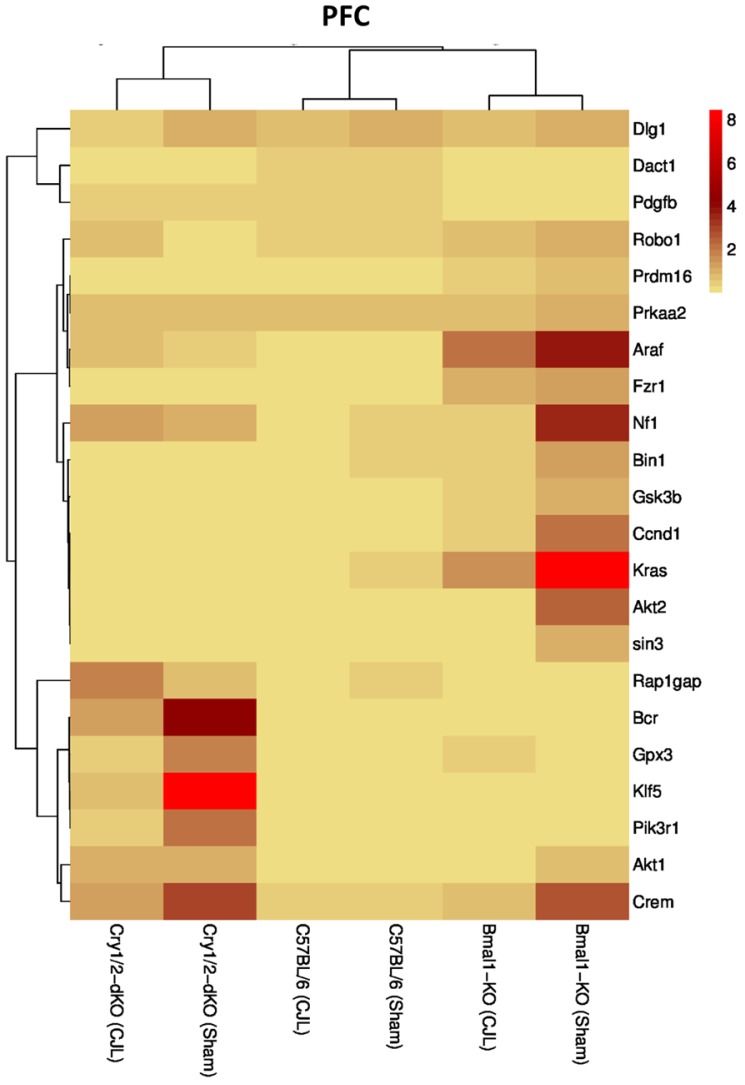
Comparative expression analysis of cancer-related genes in PFC. This figure represents the expression levels of candidate cancer genes in clock gene mutant mice in comparison with mice with the intact clock. The results represented here show fold change values of qPCR analysis. The values were normalized to the selected values of one group per brain region.

**Figure 11:**
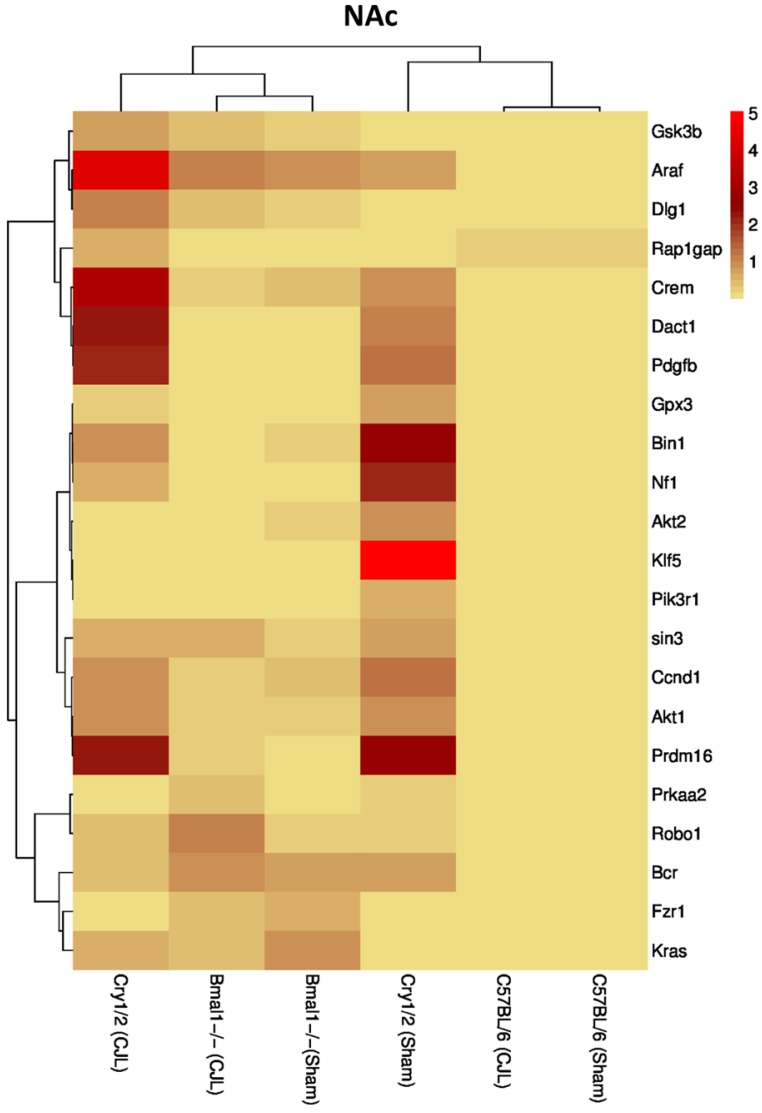
Comparative expression analysis of cancer-related genes in NAc. This figure represents the expression levels of candidate cancer genes in clock gene mutant mice in comparison with mice with the intact clock. The results represented here show fold change values of qPCR analysis. The values were normalized to the selected values of one group per brain region.

**Figure 12:**
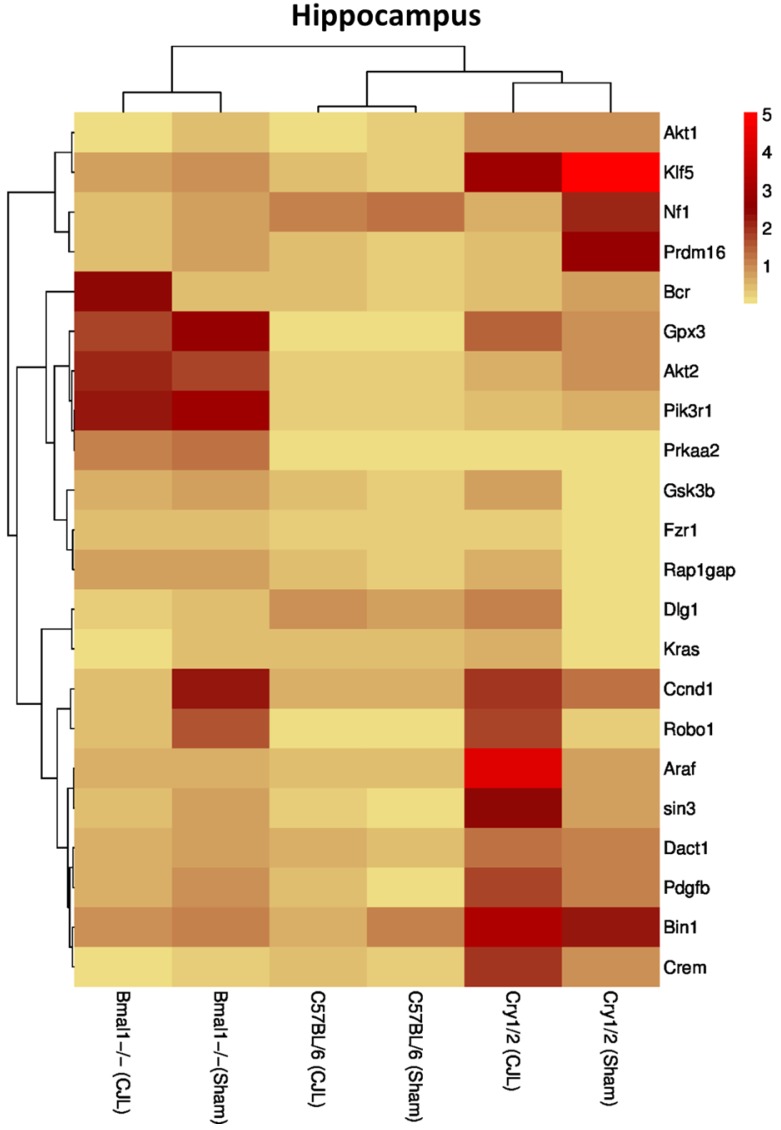
Comparative expression analysis of cancer-related genes in the hippocampus. This figure represents the expression levels of candidate cancer genes in clock gene mutant mice in comparison with mice with the intact clock. The results represented here show fold change values of qPCR analysis. The values were normalized to the selected values of one group per brain region.

**Figure 13:**
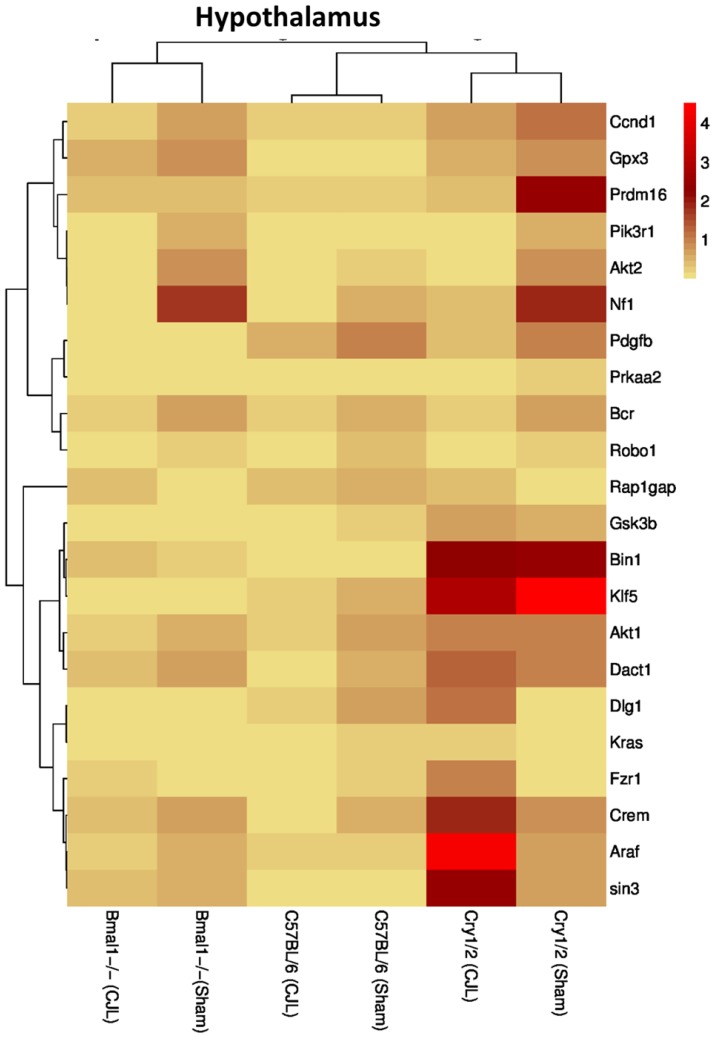
Comparative expression analysis of cancer-related genes in the hypothalamus. This figure represents the expression levels of candidate cancer genes in clock gene mutant mice in comparison with mice with the intact clock. The results represented here show fold change values of qPCR analysis. The values were normalized to the selected values of one group per brain region.

**Figure 14:**
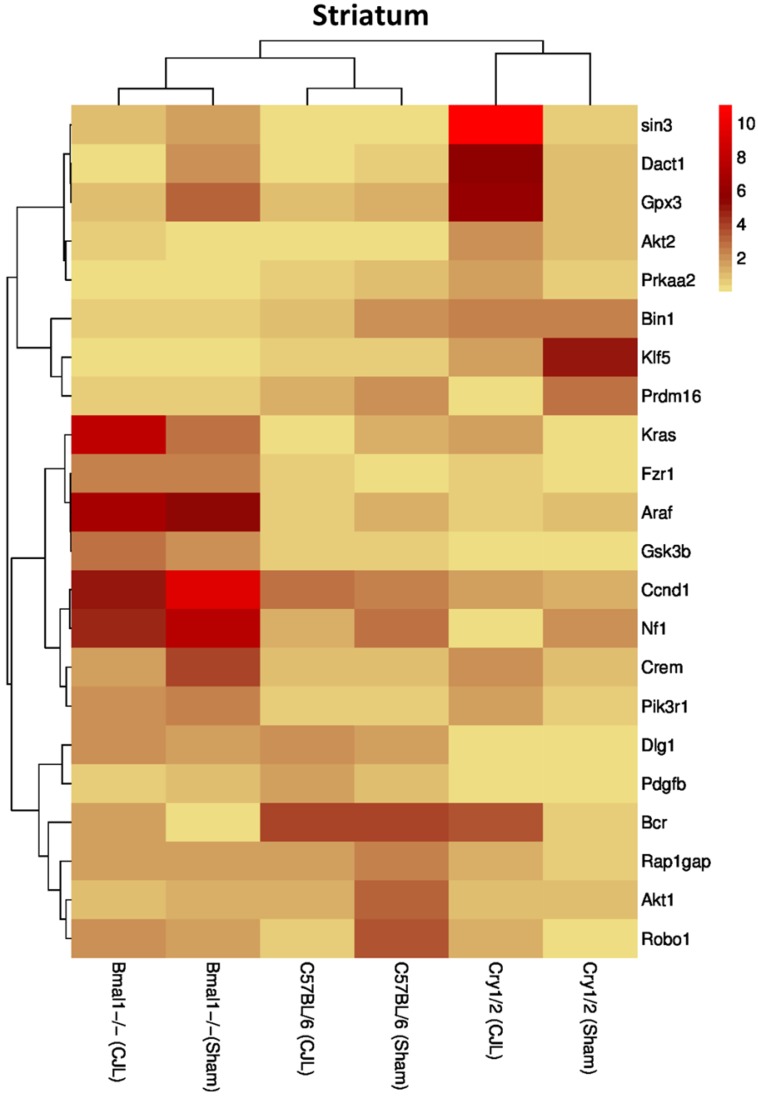
Comparative expression analysis of cancer-related genes in the striatum. This figure represents the expression levels of candidate cancer genes in clock gene mutant mice in comparison with mice with the intact clock. The results represented here show fold change values of qPCR analysis. The values were normalized to the selected values of one group per brain region.

We further investigated the association of candidate cancer-related genes clock (*Bmal1* and *Cry1/2*) genes. We compared expression levels of candidate cancer genes from *Bmal^-/-^* and *Cry1/2* knock mice with C57BL/6 mice. This analysis revealed that some of the tumor suppressor and oncogenes were differentially regulated, such as *Kras* was upregulated in PFC of *Bmal^-/-^* at baseline line level. Similarly, *Klf5* was upregulated in *Cry1/2* knockout mice. Most of the genes showed similar regulation when compared at a baseline level and post CJL level (Table S1). Overall, the genes were profoundly affected by CJL in *Bmal^-/-^* when compared sham group (Figure 10-14).

## Discussion

Complex body function regulation and optimization need a time frame clock 44. Cancer, the second leading cause of death around the globe, is linked with the environmentally disrupted circadian clock, and this link is supported by pre-clinical data 7,17,18. In the current study, we observed significant variations in the expression of several glioma-related genes in the different brain regions of wild type and clock gene mutant mice on CJL exposure. Increasing evidence by epidemiological studies strongly suggests a relationship between abnormal circadian rhythms through shiftwork and the initiation and progression of cancer 7,17,18, whereas the experimental studies support an association between clock genes and increased risk of cancer in animal models 12,45,46. However, the precise connections between chronic jetlag and expression pattern of genes associated with glioma risks in brain regions, are yet to be understood to minimize the risks and develop treatment strategies. To our knowledge, this first study was designed to investigate alterations in the expression of glioma-related genes in response to CJL in the hypothalamus, hippocampus, striatum, nucleus NAc, and prefrontal cortex using wild type, *Bmal1*^-/-^ knockout, and *Cry1/2* double knockout mice. Our results demonstrate that CJL exposure in mouse brain significantly alters the expression of genes associated with cancer prognosis and tumor suppression. In previous studies, the abnormal regulation of *Bmal* and other core clock genes have shown an increase in the risk of different types of cancers 45,47-49.

Similarly, the expression of cancer-linked genes also altered in response to environmental alteration 44,50. Our findings support the concept that CJL in the brain induces an alteration in the glioma-linked gene expression, which could cause an increase in cell proliferation 50,51. The genes we studied in our models have broad cellular functions and are strongly associated with glioma risks, specifically, glioma (Table S3). The up and downregulations of these genes may not indeed indicate cancer progression or development. However, the links of these genes to CJL in the brain provides evidence of a potential mechanism through which CJL might ultimately contribute to glioma.

Glioma and other brain tumors are usually considered a disease of deliberate death, where the average survival is maximum few years after diagnosis 52-55. Due to the direct interaction of altered light-dark cycles with the circadian clock, shiftwork or jetlag like conditions may increase the risks in developing gliomas. Several studies have demonstrated the increased risks of different cancers associated with abnormal circadian rhythms 18,20,56,57. For instance, *Bmal1* gene regulates the molecular system to prevent cancer progression in several tissues 47,49. Our data demonstrate aberrant expression of cancer-related genes in the brain regions of wild type and clock gene mutant mice in response to CJL. Therefore, CJL may either cause localized cell proliferation during the initial exposures or aggravate pre-existing neoplastic lesions.

Abnormal circadian rhythms can downregulate immune function, which in turn can impair the immune response against tumor cells 58,59. Similarly, clock genes disruption can facilitate the dissemination of tumor cells in peripheral body parts 60. However, only the downregulated immune function cannot account for cancer progression. It may also include the signals triggered by brain regions (hypothalamus and hippocampus) that may impact the synthesis of mitogenic factors and neurotransmitter release to promote tumorigenesis by acting directly on receptors present in cancer cells. They also suppress hormonal pulsatility, which can assist cell proliferation 50,53,60,61. In the case of our study, the decreased mRNA levels of tumor suppressor genes provide evidence that CJL is inducing the progression of glioma in the brain.

The expression analysis results indicated alterations in cancer-related genes in wild type mice exposed to CJL. In NAc *Crem, Robo1, Bcr,* and *Kras* were downregulated whereas, *Gpx3* was upregulated. In hippocampus *Bin1, Bcr,* and* Nf1* were downregulated, and *Crem* was upregulated. In PFC *Bin1, Bcr, Nf1,* and *Kras* were downregulated whereas *Crem* and *Araf* were upregulated. In hypothalamus *Crem, Bcr, Nf1,* and* Akt2* were downregulated. In striatum *Robo1,* and *Dact1* were downregulated while *Kras* was upregulated. The expression analysis results indicated alterations in cancer-related genes in *Bmal^-/-^* mice exposed to CJL.

In NAc *Crem, Bin1, Akt2, Dact1, Ccnd1, Fzr1,* and *Klf5* were downregulated, whereas *Robo1, Prdm16,* and *Prkaa2* were upregulated. In hippocampus *Crem, Akt1, Ccnd1, Kras,* and *Prdm16* were downregulated whereas *Bcr* was upregulated. In PFC *Crem, Nf1, Akt2, Ccnd1, Kras, Prdm16, Klf5,* and *Tspan32* were downregulated. In the hypothalamus, *Robo1, Prkaa2, Klf5,* and* Rap1gap* were upregulated. In the striatum, *Crem, Dact1, Gpx3, Ccnd1, Kras,* and *Prdm16* were downregulated whereas *Bcr* and *Klf5* were upregulated. The expression analysis results indicated alterations in cancer-related genes in *Cry1/2* double knockout mice exposed to CJL. In NAc*, Bin1, Gpx3, Bcr*, *Akt2, Kras, Prdm16, Gsk3b,* and *Pik3r1* were downregulated whereas *Nf1, Araf, Dlg1, Klf5,* and *Rap1gap* were upregulated. In the hippocampus, *Bcr, Kras* and *Prdm16* were downregulated while *NF1, Araf, Dlg1, Klf5,* and* Rap1gap* were upregulated. In PFC *Bcr,* and *Gsk3b* were downregulated whereas *Araf* and *Rap1gap* were upregulated. In the hypothalamus, *Kras, Prdm16* and *Prkaa2* were downregulated, whereas *Araf, Dlg1, Klf5,* and *Rap1gap* were upregulated. In the striatum, *Gsk3b* was downregulated whereas *Dact1, Bcr, Gpx3, Klf5, Rap1gap,* and *Tspan32* were upregulated. *Crem* (NAc)*, Bcr* (hippocampus)*, Nf1* and *Kras* (PFC), and Dact1 (striatum) were similarly regulated in wild type and *Bmal1^-/-^* mice. *Bcr* and *Kras* (NAc), and *Bcr* (hippocampus and PFC) were similarly regulated in wild type and *Cry1/2* double knockout mice. Bin1 and Akt2 (NAc), *Kras* and *Prdm16* (hippocampus), *Klf5* and *rap1gap* (hypothalamus), and *Bcr* and *Klf5* (striatum) were similarly regulated in *Bmal1^-/-^* and *Cry1/2* double knockout mice.

The expression of Crem, Dact1, Ccnd1, Fzr1, Robo1, Prdm16, Prkaa2, Gpx3, Bcr, Kras, Gsk3b, Pik3r1, Nf1, Araf, Dlg1, Klf5, and Rap1gap was inconsistent in NAc, Crem, Akt1, Ccnd1, Bcr, Nf1, Araf, Dlg1, Klf5 and Rap1gap in hippocampus, Crem, Nf1, Akt2, Ccnd1, Kras, Prdm16, Klf5, Tspan32 Bcr, Gsk3b, Araf and Rap1gap in PFC, Robo1, Kras, Prdm16 and Prkaa2, Araf, Dlg1, in hypothalamus, and Gsk3b Dact1, Gpx3, Rap1gap and Tspan32 of clock gene mutant mice. As the expression level of glioma-related genes was found differentially expressed in the comparison between WT, *Bmal1^-/-^* and *Cry1/2*-KO knockout mice, we suggest that clock genes may have a direct or indirect association with expression patterns of these genes (Table 1). For instance, the upregulation of *Crem* in *Bmal1^-/-^* mice in PFC suggests that its expression is associated with *Bmal1* directly or indirectly as this gene was downregulated in PFC of wild type mice.

Furthermore, *Cry1/2*-KO the gene was found unaltered suggesting that the *Crem*'s expression is dependent on *Cry1/2* gene/s. *Crem* has been previously implicated as a key player in tumor regulation/suppression 62,63, hence its altered expression also indicates that CJL can increase cancer risk if *Bmal1* is nonfunctional while the non-functionality of *Cry1/2* has an opposite effect. Similarly, lacking functional *Bmal1*may increase the CJL mediated glioma risk via downregulating the previously reported tumor suppressor genes *Bin1*^53^,* Tspan32* 64,65*, Dact1* 66,67*, Fzr1* 68*, Gpsx3* 55,69*,* and *Gsk3b* 70, whereas lacking functional *Cry1/2*, may play a role in preventing CJL mediated cancer via downregulating the oncogenes *Akt1* 71,72*, Kras* 73,74*, Pik3r1* (75)and *Prdm* 76,77, which have been reported as oncogenes that promote cancer metastasis and proliferation.

In contrast, the presence of functional *Cry1/2* and *Bmal1*may play a role in preventing CJL mediate cancer development by interacting with cancer-associated genes that were reported playing role in cancer including *Tspan32* 64,65*, Rap1gap* 78*, Prkaa2* 79,80*, Fzr1*^68^*, Gsk3b* 70*, Dlg1*^61^*, Klf5* 81,82*, Akt1* 72,79,83*, Araf* 84*, Ccnd1*^85^*, Pik3r1*^75^ and *Prdm16* 76,77.

Our results indicated that the tumor suppressor genes were downregulated, whereas the oncogenes were upregulated in WT mice, unlike the clock genes mutant mice. Although, some genes were regulated in a similar pattern such as *Kras*, *Rap1gap, Araf,* and *Dlg1* were upregulated only. In contrast, *Bin1, Fzr1, Akt1, Akt2*, and *Pik3r1* were downregulated only. Despite the lack of knowledge, several studies have identified multiple molecular and physiological pathways that affect the circadian clock system and thus leads to cancer progression 18,49,86.

The genes found altered in this report may be tissue-specific, as evident from the different genes found upregulated in one region while unaltered and downregulated in another area on CJL treatment. For instance, *Bcr* was downregulated in PFC and hippocampus while upregulated in the striatum. We detected that *Bmal1* has a direct link with tumor invasion in the brain through interaction with *Mdm2* and other cancer-related genes (Figure 3). *Mdm2* is a proto-oncogene that plays an essential role in human sarcomas, which is overexpressed in a wide variety of cancers. Its protein forms a complex with the p53 protein. Hence, its oncogenic potential is p53-dependent 87.

Considering the importance of the *Bmal1* gene in cancer prevention and its association with the clock system, we investigated the expression of tumor suppressor genes to determine if *Bmal^-/-^*differentially express these genes. In wild type mice (on CJL treatment), *Crem* downregulated in NAc and hypothalamus, *Bin1* in PFC and hippocampus, *Robo1* in NAc and striatum, *Dact1* in the striatum, *Bcr* in NAc, hippocampus, and PFC, and *Nf1* in the hippocampus, PFC and hypothalamus. Similarly, in Bmal^-/-^ knockout mice, *Bin1* was downregulated in NAc, *Bcr* was downregulated in NAc, hippocampus, *Prkaa2* in the hypothalamus, and *Gpx3* in NAc. In contrast, *Dact1, Bcr,* and *Gpx3* were upregulated in the striatum, *Nf1* in NAc and hippocampus, *Dlg1* in NAc, hippocampus, and hypothalamus, *Klf5* in NAc, hippocampus, hypothalamus, and striatum, *Rap1gap* in NAc, hippocampus, PFC, hypothalamus, and striatum and *Tspan32* in the striatum.

In our study, *Prkaa2* and *Ccnd1,* and *Nf1* showed upregulation in *Bmal1^-/-^*mice and downregulation in *Cry1/2*-KO mice and vice versa. These genes are linked with cancer 79,85,88. This regulation suggests a relationship between Cry*1/2* and *Bmal1* and *Prkaa2, Ccnd1*and *Nf1* in promoting CJL mediated cancer. CJL can significantly alter the expression of multiple genes affecting several molecular functions leading to increased cell proliferation, mitogenic signaling, altered bioenergetics, apoptosis, DNA repair, and extracellular matrix. Furthermore, cancer is a multistep and highly complex process. CJL alone cannot provide all the specific characteristics for cellular proliferation and transformation into a malignant phenotype. However, it may dysregulate certain pro- and anti-proliferative cellular processes. We observed CJL induce upregulation of oncogenes and downregulation of TSGs. Our findings are in support of the previous findings that shiftwork increases cancer risks 19,22. In an earlier study, *Bmal1* has been found associated with tumor suppression 47; hence its non-functionality may upregulate oncogenes. However, these findings contradict with our gene expression data. The observed higher expression of TSGs in case of *Bmal1^-/-^* mouse could be a compensatory response by other clock genes or anticancer enzymes. The brain is sensitive to signals that affect tumorigenic growth, and therefore, sustains a cell death/proliferation equilibrium 52,85. The upregulated expression of oncogenes and downregulation of TSGs is noteworthy.

Further analysis of tumor suppressor and oncogenes in C57BL/6 mice compared with *Bmal1^-/-^* and *Cry1/2* mice showed significant differences among mRNA levels of several genes specific to brain regions. These differences indicated that clock genes are directly linked with glioma risk. The present study has some limitations. First, the tissues were dissected following standard protocols. However, being small size and hard to separate closely adjacent tissues, it might be possible that this surrounding may have had residual cells with the processed regions although brain regions we used were well isolated from other tissues. To reduce contamination, we dissected using microdissection. Secondly, we examined only male mice (*Bmal1^-/-^* and *Cry1/2*-KO) while female mice can also be considered in future experiments.

## Conclusion

This study illustrates the effects of the disrupted circadian clock via CJL on the expression of cancer-related genes in 5 different brain regions in wild type and clock mutant male mice. As indicated by the functions and related molecular pathways, the candidate genes were found mainly associated with glioma. CJL may be an early stimulus that induces glioma and other cancers by influencing circadian clock that may, in turn, affects a wide variety of cellular functions. These results suggest the association of clock genes (*Bmal1*, *Cry1,* and *Cry2*) with the modification in the expression of glioma-related genes. The intact circadian clock resists the alteration in expression of some glioma promoting genes, but not others, provide support for future investigations related to glioma diagnosis, prognosis, and treatment in association with light-dark cycles regulation and a circadian clock function.

## Supplementary Material

Supplementary figures and tables.

## Figures and Tables

**Table 1:** List of primers

GENE	FORWARD PRIMER SEQUENCE	REVERSE PRIMER SEQUENCE
*Crem*	5'ACTCTAGCTCAGGTAGCAACA 3'	5'AGGTGGTGTCCCTTCTTCCT 3'
*Tspan32*	5'ACTTCCTAGTCTTGCTGCTGG 3'	5'GCCCAATAGCGCAGTGTTTC 3'
*Bin1*	5'TTGCCAAGGCAGAAGAGGAG 3'	5'CTCCTGCAGATCCACGTTCA 3'
*Robo1*	5'GCTGGCGACATGGGATCATA 3'	5'AATGTGGCGGCTCTTGAACT 3'
*Dact1*	5'CGGCCTAGCTCAGGGTTTTA 3'	5'CCGCCTTTACATTCCAACCA 3'
*Rap1gap*	5'GCTTCACCTTCGGTGCCTAT 3'	5'CCCATCACTCCTCCACACAC 3'
*Bcr*	5'CAGAATTCGCAGCAGTCCTTT 3'	5'TGTTCCAAACGAGGAATCTGCT 3'
*Prkaa2*	5'GGCAAAGTGAAGACTACCAGG 3'	5'TGTGACAGTAATCCACGGCA 3'
*Fzr1*	5'CGTGACCGCATGATCCTACA 3'	5'ACACGAGCAGCTTGTTGTCA 3'
*Gpx3*	5'TTCCTGAAGAACTCCTGCCC 3'	5'GTTCCAGCGGATGTCATGGA 3'
*Gsk3b*	5'GCATTTATCATTAACCTAGCACCCT 3'	5'GCTGCCATCTTTATCTCTGCTA 3'
*Nf1*	5'AGTGAAAGTAGTTACCGTGGTC 3'	5'CCTCATAGTCACGCTTCGGT 3'
*Dlg1*	5'CTCTGGTCATCAGTGGGCTC 3'	5'CTTTGGTTGCCCAGCAAGAC 3'
*Klf5*	5'AGCTGGTCCAGACAAGATGTG 3'	5'ACTGGTCTACCACTGAGGCA 3'
*Akt1*	5'TGAGAAGAAGCTGAGCCCAC 3'	5'TAGGAGAACTTGATCAGGCGG 3'
*Araf*	5'AACCGCCGACAGTTCTACC 3'	5'CCCTGGCCTTTCATCTACGA 3'
*Akt2*	5'CTGACTCCGAGAAGGCGTC 3'	5'CAGTATCGTCTGTCACCGGC 3'
*Ccnd1*	5'TCAAGTGTGACCCGGACTG 3'	5'GATGTCCACATCTCGCACG 3'
*Kras*	5'TGAAGATGTGCCTATGGTCCTG 3'	5'GCATCGTCAACACCCTGTCT 3'
*Pdgfb*	5'GCTCCGTCTACGCGTCC 3'	5'GAATGGGATCCCCCTCGG 3'
*Pik3r1*	5'CACCATTACAAAGAAAGCCGGA 3'	5'GGGCAGTGCTGGTGGAT 3'
*Prdm16*	5'GAAGTCACAGGAGGACACGG 3'	5'TCATTGCATATGCCTCCGGG 3'

**Table 2:** Final selected genes associated with glioma

Gene symbol	Ensemble ID	Mean expression in brain	highest expression time-point	lowest expression time-point
Tumor suppressor genes				
*Crem*	ENSMUSG00000063889	4.410036144	ZT2	ZT14
*Tspan32*	ENSMUSG00000000244	5.689827594	ZT6	ZT14
*Bin1*	ENSMUSG00000024381	4.722469534	ZT6 and 10	ZT22
*Robo1*	ENSMUSG00000022883	5.971814277	ZT6 and 14	ZT2, 10 and 18
*Dact1*	ENSMUSG00000044548	4.794540791	ZT10	ZT2 and 14
*Rap1gap*	ENSMUSG00000041351	7.611098895	ZT14	ZT2 and ZT22
*Bcr*	ENSMUSG00000009681	9.403224037	ZT4	ZT6 and 10
*Prkaa2*	ENSMUSG00000028518	8.473877937	ZT6	ZT18
*Fzr1*	ENSMUSG00000020235	7.298708704	ZT10	ZT22
*Gpx3*	ENSMUSG00000018339	2.796361742	ZT10	ZT22
*Gsk3b*	ENSMUSG00000022812	2.892956828	ZT2	ZT18
*Nf1*	ENSMUSG00000020716	5.168002969	ZT6	ZT22
*Dlg1*	ENSMUSG00000022770	4.647283501	ZT2	ZT10
*Klf5*	ENSMUSG00000005148	4.792437773	ZT10	ZT2 and 22
Oncogenes				
*Akt1*	ENSMUSG00000001729	2.866842546	ZT10	ZT22,2
*Akt2*	ENSMUSG00000004056	5.15949289	ZT14	ZT22,2
*Araf*	ENSMUSG00000001127	9.892118727	ZT2,22	ZT14
*Ccnd1*	ENSMUSG00000070348	4.013195242	ZT6	ZT14
*Kras*	ENSMUSG00000030265	3.594204131	ZT2,6,10,22	ZT14,18
*Pdgfb*	ENSMUSG00000000489	3.809463804	ZT14	ZT22,2
*Pik3r1*	ENSMUSG00000041417	6.597822674	ZT2-14,22	ZT14
*Prdm16*	ENSMUSG00000039410	9.218920632	ZT6,18	ZT10,22

## Abbreviations

CJL: Chronic jetlag
*Bmal1*: Brain and muscle ARNT like protein 1
*Cry1/2*: Cryptochrome ½
TSGs: Tumor suppressor genes
NAc: Nucleus Accumbens
PFC: Prefrontal cortex
