# Supplementary material for: Impact of chronically alternating light-dark cycles on circadian clock mediated expression of cancer (glioma)-related genes in the brain
Source: Int J Biol Sci. 2019 Jul 20;15(9):1816–34. doi: 10.7150/ijbs.35520 (PMC6743288; doi:10.7150/ijbs.35520)
Supplement: Supplementary file 1 — Supplementary figures and tables. [file ijbsv15p1816s1.pdf]

Table S1. Expression levels of mRNA in wild type and clock gene mutant mice compared with each other. T-test represents correlation among groups: (T-test-1)- C57BL/6 *vs* Bmal1$^{-/-}$, (T-test-2)- C57BL/6 *vs* *Cry1/2*, (T-test-3)- Bmal1$^{-/-}$ *vs* *Cry1/2*.

CJL group						
**Genes**	**C57BL/6 ± SEM**	**T-test-1**	**Bmal1-/- ± SEM**	**T-test-2**	**Cry1/2 ± SEM**	**T-test-3**
**PFC**						
Akt1	0.073 ± 0.031	0.201	0.180 ± 0.058	0.001	1.000 ± 0.000	0.005
Akt2	0.145 ± 0.033	0.640	0.173 ± 0.044	0.281	0.096 ± 0.020	0.214
Araf	0.181 ± 0.037	0.068	2.131 ± 0.541	0.002	0.822 ± 0.009	0.136
Bcr	0.192 ± 0.068	0.537	0.139 ± 0.030	0.001	1.337 ± 0.041	0.001
Bin1	0.201 ± 0.048	0.093	0.411 ± 0.076	0.225	0.118 ± 0.029	0.047
Ccnd1	0.242 ± 0.059	0.159	0.411 ± 0.076	0.716	0.214 ± 0.044	0.105
Crem	0.465 ± 0.108	0.312	0.734 ± 0.195	0.097	1.155 ± 0.253	0.262
Dact1	0.315 ± 0.082	0.492	0.240 ± 0.052	0.085	0.061 ± 0.016	0.065
Dlg1	0.635 ± 0.143	0.740	0.703 ± 0.127	0.343	0.459 ± 0.014	0.193
Fzr1	0.049 ± 0.014	0.062	0.950 ± 0.237	0.880	0.052 ± 0.003	0.063
Gpx3	0.049 ± 0.014	0.077	0.331 ± 0.086	0.180	0.321 ± 0.135	0.950
Klf5	0.082 ± 0.032	0.336	0.213 ± 0.104	0.009	0.764 ± 0.087	0.016
Kras	0.006 ± 0.001	0.047	1.611 ± 0.360	0.029	0.082 ± 0.014	0.051
Nf1	0.069 ± 0.024	0.072	0.367 ± 0.090	0.114	1.186 ± 0.415	0.182
Gsk3b	0.036 ± 0.005	0.054	0.355 ± 0.078	0.975	0.036 ± 0.008	0.053
Pdgfb	0.312 ± 0.089	0.664	0.253 ± 0.088	0.871	0.295 ± 0.036	0.697
Pik3r1	0.086 ± 0.036	0.192	0.016 ± 0.004	0.047	0.408 ± 0.085	0.044
Prdm16	0.037 ± 0.009	0.040	0.442 ± 0.085	0.012	0.284 ± 0.033	0.197
Prkaa2	0.571 ± 0.307	0.617	0.760 ± 0.141	0.826	0.679 ± 0.345	0.844
Rap1gap	0.188 ± 0.047	0.472	0.141 ± 0.035	0.001	1.837 ± 0.036	0.000
Robo1	0.305 ± 0.091	0.155	0.722 ± 0.196	0.236	0.662 ± 0.216	0.847
sin3	0.104 ± 0.025	0.229	0.191 ± 0.052	0.253	0.065 ± 0.009	0.131
**NAc**						
Akt1	0.021 ± 0.004	0.039	0.235 ± 0.044	0.001	1.000 ± 0.000	0.003
Akt2	0.030 ± 0.002	0.366	0.040 ± 0.009	0.174	0.081 ± 0.025	0.239
Araf	0.043 ± 0.007	0.058	1.073 ± 0.260	0.019	4.263 ± 0.584	0.019
Bcr	0.078 ± 0.017	0.052	0.877 ± 0.192	0.020	0.498 ± 0.069	0.179
Bin1	0.046 ± 0.021	0.281	0.081 ± 0.018	0.007	0.954 ± 0.088	0.008
Ccnd1	0.027 ± 0.013	0.063	0.244 ± 0.060	0.006	0.873 ± 0.072	0.003
Crem	0.036 ± 0.007	0.060	0.242 ± 0.054	0.009	3.287 ± 0.313	0.009
Dact1	0.032 ± 0.002	0.049	0.018 ± 0.004	0.014	2.340 ± 0.273	0.014
Dlg1	0.153 ± 0.011	0.122	0.398 ± 0.095	0.014	1.115 ± 0.118	0.010
Fzr1	0.063 ± 0.012	0.062	0.471 ± 0.108	0.842	0.060 ± 0.003	0.062
Gpx3	0.054 ± 0.011	0.075	0.153 ± 0.032	0.008	0.313 ± 0.031	0.023

Klf5	0.127	±	0.025	0.039	0.007	±	0.002	0.198	0.082	±	0.008	0.008
Kras	0.012	±	0.001	0.064	0.482	±	0.128	0.007	0.642	±	0.055	0.340
Nf1	0.034	±	0.010	0.126	0.079	±	0.019	0.022	0.555	±	0.082	0.023
Gsk3b	0.074	±	0.012	0.066	0.476	±	0.110	0.026	0.744	±	0.112	0.164
Pdgfb	0.098	±	0.016	0.718	0.088	±	0.020	0.005	2.077	±	0.140	0.004
Pik3r1	0.061	±	0.019	0.778	0.055	±	0.013	0.167	0.102	±	0.014	0.067
Prdm16	0.030	±	0.021	0.051	0.316	±	0.074	0.019	2.207	±	0.310	0.021
Prkaa2	0.053	±	0.014	0.049	0.467	±	0.098	0.227	0.119	±	0.039	0.056
Rap1gap	0.324	±	0.112	0.206	0.120	±	0.027	0.146	0.594	±	0.100	0.034
Robo1	0.044	±	0.015	0.051	1.109	±	0.252	0.001	0.375	±	0.013	0.100
sin3	0.066	±	0.029	0.042	0.564	±	0.114	0.001	0.545	±	0.033	0.887
**Hippocampus**												
Akt1	0.115	±	0.029	0.539	0.089	±	0.024	0.001	1.000	±	0.000	0.002
Akt2	0.182	±	0.088	0.045	2.111	±	0.446	0.023	0.625	±	0.040	0.078
Araf	0.514	±	0.198	0.779	0.589	±	0.147	0.016	4.263	±	0.584	0.019
Bcr	0.358	±	0.030	0.059	2.417	±	0.526	0.168	0.498	±	0.069	0.065
Bin1	0.654	±	0.167	0.357	0.923	±	0.196	0.070	3.288	±	0.773	0.084
Ccnd1	0.665	±	0.183	0.388	0.457	±	0.097	0.038	1.998	±	0.335	0.036
Crem	0.445	±	0.113	0.083	0.086	±	0.019	0.004	1.888	±	0.180	0.009
Dact1	0.677	±	0.187	0.739	0.595	±	0.126	0.054	1.344	±	0.157	0.022
Dlg1	0.867	±	0.226	0.128	0.322	±	0.068	0.403	1.115	±	0.118	0.009
Fzr1	0.293	±	0.124	0.290	0.494	±	0.107	0.899	0.276	±	0.014	0.176
Gpx3	0.038	±	0.010	0.050	1.822	±	0.416	0.010	1.436	±	0.141	0.457
Klf5	0.415	±	0.150	0.242	0.745	±	0.186	0.004	3.006	±	0.288	0.005
Kras	0.438	±	0.115	0.082	0.068	±	0.015	0.212	0.642	±	0.055	0.006
Nf1	1.093	±	0.276	0.143	0.473	±	0.096	0.236	0.638	±	0.094	0.288
Gsk3b	0.395	±	0.163	0.395	0.592	±	0.123	0.162	0.744	±	0.112	0.413
Pdgfb	0.376	±	0.296	0.544	0.608	±	0.177	0.079	1.707	±	0.450	0.121
Pik3r1	0.256	±	0.047	0.086	2.282	±	0.639	0.060	0.466	±	0.063	0.103
Prdm16	0.376	±	0.100	0.973	0.381	±	0.083	0.580	0.448	±	0.063	0.555
Prkaa2	0.068	±	0.019	0.294	1.042	±	0.691	0.451	0.087	±	0.012	0.301
Rap1gap	0.471	±	0.153	0.415	0.737	±	0.242	0.543	0.594	±	0.100	0.629
Robo1	0.159	±	0.043	0.072	0.514	±	0.112	0.006	1.722	±	0.059	0.002
sin3	0.247	±	0.065	0.445	0.462	±	0.225	0.001	2.504	±	0.151	0.003
**Hypothalamus**												
Akt1	0.293	±	0.077	0.242	0.169	±	0.029	0.012	1.000	±	0.000	0.001
Akt2	0.049	±	0.013	0.273	0.071	±	0.011	0.861	0.053	±	0.017	0.446
Araf	0.212	±	0.086	0.602	0.266	±	0.037	0.018	4.263	±	0.584	0.020
Bcr	0.264	±	0.080	0.738	0.232	±	0.032	0.315	0.159	±	0.022	0.140
Bin1	0.032	±	0.008	0.130	0.433	±	0.161	0.228	2.367	±	1.360	0.290
Ccnd1	0.161	±	0.042	0.074	0.288	±	0.024	0.078	0.663	±	0.159	0.139
Crem	0.081	±	0.021	0.093	0.314	±	0.080	0.009	1.888	±	0.180	0.005
Dact1	0.054	±	0.014	0.011	0.336	±	0.039	0.014	1.344	±	0.157	0.018
Dlg1	0.292	±	0.076	0.098	0.070	±	0.007	0.007	1.115	±	0.118	0.012

Fzr1	0.106	±	0.049	0.144	0.229	±	0.047	0.422	1.92	±	1.28	0.423
Gpx3	0.013	±	0.003	0.417	0.574	±	0.553	0.401	0.590	±	0.545	0.985
Klf5	0.252	±	0.002	0.001	0.047	±	0.005	0.011	3.006	±	0.288	0.009
Kras	0.088	±	0.023	0.484	0.068	±	0.006	0.427	0.303	±	0.218	0.393
Nf1	0.112	±	0.029	0.268	0.068	±	0.010	0.478	0.327	±	0.249	0.407
Gsk3b	0.098	±	0.015	0.053	0.046	±	0.007	0.027	0.744	±	0.112	0.025
Pdgfb	0.512	±	0.088	0.038	0.075	±	0.005	0.368	0.330	±	0.151	0.232
Pik3r1	0.023	±	0.001	0.050	0.051	±	0.007	0.073	0.041	±	0.006	0.344
Prdm16	0.208	±	0.054	0.077	0.390	±	0.055	0.162	0.330	±	0.046	0.453
Prkaa2	0.045	±	0.012	0.095	0.009	±	0.001	0.088	0.008	±	0.001	0.348
Rap1gap	0.320	±	0.109	0.385	0.451	±	0.076	0.605	0.392	±	0.066	0.592
Robo1	0.093	±	0.024	0.974	0.094	±	0.003	0.334	0.124	±	0.004	0.006
sin3	0.018	±	0.005	0.062	0.426	±	0.107	0.004	2.504	±	0.151	0.001
**Striatum**												
Akt1	1.143	±	0.251	0.891	1.092	±	0.246	0.626	1.000	±	0.000	0.745
Akt2	0.059	±	0.013	0.049	0.635	±	0.134	0.237	2.204	±	1.284	0.346
Araf	0.511	±	0.113	0.041	6.982	±	1.368	0.168	0.742	±	0.045	0.045
Bcr	3.843	±	0.840	0.118	1.687	±	0.181	0.792	3.371	±	1.417	0.356
Bin1	0.809	±	0.161	0.782	0.743	±	0.153	0.114	2.550	±	0.667	0.107
Ccnd1	2.759	±	0.612	0.183	5.052	±	1.180	0.325	1.706	±	0.710	0.086
Crem	1.005	±	0.220	0.220	1.559	±	0.304	0.402	2.031	±	0.971	0.682
Dact1	0.115	±	0.026	0.971	0.114	±	0.023	0.072	5.677	±	1.583	0.072
Dlg1	1.974	±	0.868	0.834	2.200	±	0.485	0.173	0.170	±	0.045	0.051
Fzr1	0.586	±	0.128	0.060	2.404	±	0.501	0.355	0.432	±	0.050	0.057
Gpx3	0.774	±	0.171	0.436	0.999	±	0.195	0.080	6.098	±	1.624	0.086
Klf5	0.618	±	0.141	0.054	0.044	±	0.011	0.153	1.604	±	0.453	0.075
Kras	0.318	±	0.070	0.057	7.841	±	1.875	0.098	1.581	±	0.436	0.072
Nf1	1.482	±	0.344	0.082	4.630	±	1.038	0.071	0.329	±	0.084	0.053
Gsk3b	0.597	±	0.139	0.057	2.637	±	0.546	0.058	0.059	±	0.018	0.042
Pdgfb	1.710	±	0.386	0.087	0.566	±	0.115	0.049	0.040	±	0.012	0.044
Pik3r1	0.491	±	0.164	0.052	2.157	±	0.447	0.176	1.764	±	0.634	0.641
Prdm16	1.161	±	0.274	0.115	0.472	±	0.099	0.071	0.231	±	0.056	0.120
Prkaa2	0.491	±	0.260	0.622	0.339	±	0.068	0.177	1.568	±	0.545	0.151
Rap1gap	1.850	±	1.018	0.803	1.553	±	0.305	0.694	1.377	±	0.302	0.704
Robo1	0.615	±	0.134	0.061	2.048	±	0.410	0.158	1.445	±	0.392	0.348
sin3	0.132	±	0.029	0.050	1.064	±	0.221	0.090	4.780	±	0.513	0.104

Sham group

**PFC**												
Akt1	0.067	±	0.011	0.088	0.582	±	0.165	0.001	1.000	±	0.000	0.126
Akt2	0.158	±	0.026	0.061	2.446	±	0.592	0.157	0.095	±	0.026	0.058
Araf	0.062	±	0.025	0.055	3.885	±	0.939	0.066	0.413	±	0.101	0.064
Bcr	0.251	±	0.043	0.727	0.282	±	0.068	0.060	4.263	±	1.033	0.061
Bin1	0.509	±	0.080	0.116	1.163	±	0.258	0.036	0.188	±	0.051	0.058
Ccnd1	0.268	±	0.063	0.068	1.994	±	0.482	0.085	0.087	±	0.027	0.058

Crem	0.313	±	0.053	0.067	2.576	±	0.624	0.159	2.841	±	1.150	0.853
Dact1	0.502	±	0.086	0.045	0.178	±	0.043	0.056	0.210	±	0.051	0.653
Dlg1	0.879	±	0.159	0.580	1.064	±	0.257	0.781	0.963	±	0.233	0.787
Fzr1	0.053	±	0.008	0.058	1.375	±	0.334	0.831	0.057	±	0.014	0.058
Gpx3	0.053	±	0.008	0.082	0.272	±	0.068	0.126	1.875	±	0.718	0.154
Klf5	0.064	±	0.011	0.225	0.196	±	0.076	0.091	8.427	±	2.707	0.093
Kras	0.301	±	0.052	0.058	8.328	±	2.016	0.037	0.068	±	0.017	0.055
Nf1	0.460	±	0.076	0.069	3.441	±	0.833	0.153	1.085	±	0.285	0.093
Gsk3b	0.054	±	0.015	0.059	0.992	±	0.240	0.482	0.040	±	0.010	0.058
Pdgfb	0.423	±	0.161	0.344	0.224	±	0.054	0.750	0.349	±	0.143	0.482
Pik3r1	0.129	±	0.050	0.897	0.146	±	0.110	0.070	2.113	±	0.560	0.067
Prdm16	0.090	±	0.014	0.067	0.830	±	0.203	0.141	0.277	±	0.080	0.097
Prkaa2	0.634	±	0.102	0.273	0.992	±	0.240	0.840	0.676	±	0.163	0.345
Rap1gap	0.498	±	0.161	0.214	0.215	±	0.052	0.484	0.681	±	0.175	0.106
Robo1	0.380	±	0.061	0.136	0.874	±	0.212	0.080	0.194	±	0.050	0.078
sin3	0.224	±	0.036	0.080	1.058	±	0.256	0.145	0.135	±	0.033	0.067
**NAc**												
Akt1	0.087	±	0.029	0.115	0.208	±	0.048	0.001	1.000	±	0.000	0.004
Akt2	0.035	±	0.001	0.031	0.209	±	0.031	0.015	0.929	±	0.112	0.018
Araf	0.038	±	0.012	0.014	1.000	±	0.120	0.032	0.807	±	0.143	0.362
Bcr	0.066	±	0.017	0.015	0.837	±	0.102	0.016	0.750	±	0.093	0.564
Bin1	0.041	±	0.006	0.025	0.245	±	0.034	0.008	2.761	±	0.241	0.008
Ccnd1	0.029	±	0.010	0.017	0.357	±	0.048	0.005	1.248	±	0.091	0.003
Crem	0.065	±	0.015	0.007	0.418	±	0.041	0.006	0.895	±	0.073	0.009
Dact1	0.073	±	0.029	0.937	0.071	±	0.010	0.002	1.035	±	0.029	0.001
Dlg1	0.124	±	0.022	0.058	0.280	±	0.046	0.235	0.088	±	0.007	0.049
Fzr1	0.059	±	0.019	0.022	0.615	±	0.090	0.443	0.083	±	0.020	0.023
Gpx3	0.022	±	0.003	0.008	0.095	±	0.009	0.003	0.740	±	0.042	0.003
Klf5	0.111	±	0.044	0.162	0.016	±	0.005	0.014	5.046	±	0.588	0.013
Kras	0.005	±	0.001	0.047	0.917	±	0.205	0.015	0.057	±	0.007	0.052
Nf1	0.058	±	0.019	0.834	0.063	±	0.010	0.002	2.116	±	0.097	0.002
Gsk3b	0.057	±	0.012	0.022	0.306	±	0.043	0.306	0.042	±	0.002	0.025
Pdgfb	0.086	±	0.016	0.914	0.089	±	0.027	0.006	1.180	±	0.030	0.001
Pik3r1	0.068	±	0.021	0.136	0.017	±	0.002	0.003	0.645	±	0.050	0.006
Prdm16	0.026	±	0.006	0.011	0.118	±	0.013	0.001	2.859	±	0.101	0.001
Prkaa2	0.046	±	0.011	0.430	0.035	±	0.004	0.112	0.229	±	0.068	0.104
Rap1gap	0.212	±	0.082	0.157	0.031	±	0.005	0.336	0.109	±	0.015	0.024
Robo1	0.058	±	0.014	0.035	0.240	±	0.041	0.014	0.255	±	0.032	0.782
sin3	0.085	±	0.034	0.012	0.278	±	0.026	0.004	0.709	±	0.069	0.015
**Hippocampus**												
Akt1	0.223	±	0.016	0.005	0.391	±	0.023	0.004	1.000	±	0.000	0.001
Akt2	0.302	±	0.056	0.034	1.779	±	0.294	0.075	0.972	±	0.208	0.096
Araf	0.466	±	0.044	0.238	0.675	±	0.128	0.130	0.807	±	0.143	0.528
Bcr	0.300	±	0.115	0.253	0.496	±	0.089	0.041	0.750	±	0.093	0.120

Bin1	1.130	±	0.083	0.709	1.034	±	0.217	0.014	2.355	±	0.195	0.011
Ccnd1	0.616	±	0.133	0.041	2.330	±	0.405	0.022	1.248	±	0.091	0.110
Crem	0.230	±	0.026	0.064	0.330	±	0.029	0.006	0.895	±	0.073	0.008
Dact1	0.418	±	0.024	0.068	0.718	±	0.089	0.001	1.035	±	0.029	0.059
Dlg1	0.810	±	0.272	0.298	0.433	±	0.051	0.117	0.088	±	0.007	0.020
Fzr1	0.260	±	0.047	0.029	0.484	±	0.048	0.047	0.083	±	0.020	0.007
Gpx3	0.014	±	0.001	0.018	2.703	±	0.364	0.017	0.955	±	0.124	0.030
Klf5	0.300	±	0.092	0.021	0.948	±	0.135	0.013	5.046	±	0.588	0.016
Kras	0.443	±	0.036	0.701	0.409	±	0.072	0.007	0.057	±	0.007	0.039
Nf1	1.194	±	0.131	0.061	0.755	±	0.102	0.006	2.116	±	0.097	0.001
Gsk3b	0.257	±	0.118	0.071	0.725	±	0.147	0.208	0.042	±	0.002	0.044
Pdgfb	0.125	±	0.052	0.015	0.945	±	0.132	0.003	1.180	±	0.030	0.211
Pik3r1	0.193	±	0.084	0.176	3.012	±	1.371	0.016	0.645	±	0.050	0.226
Prdm16	0.241	±	0.017	0.079	0.830	±	0.178	0.001	2.859	±	0.101	0.002
Prkaa2	0.035	±	0.006	0.017	1.197	±	0.155	0.050	0.095	±	0.016	0.018
Rap1gap	0.347	±	0.019	0.042	0.787	±	0.098	0.001	0.109	±	0.015	0.018
Robo1	0.038	±	0.002	0.012	1.537	±	0.164	0.021	0.255	±	0.032	0.013
sin3	0.044	±	0.004	0.012	0.763	±	0.081	0.010	0.709	±	0.069	0.639
**Hypothalamus**												
Akt1	0.722	±	0.054	0.210	0.608	±	0.054	0.035	1.000	±	0.000	0.018
Akt2	0.247	±	0.024	0.082	0.789	±	0.169	0.076	0.870	±	0.186	0.765
Araf	0.305	±	0.034	0.273	0.499	±	0.131	0.074	0.723	±	0.128	0.289
Bcr	0.578	±	0.149	0.865	0.609	±	0.075	0.621	0.671	±	0.083	0.610
Bin1	0.113	±	0.008	0.013	0.280	±	0.024	0.008	2.471	±	0.216	0.009
Ccnd1	0.210	±	0.007	0.190	0.753	±	0.278	0.008	1.117	±	0.082	0.319
Crem	0.498	±	0.061	0.526	0.612	±	0.145	0.027	0.801	±	0.065	0.326
Dact1	0.519	±	0.189	0.722	0.627	±	0.210	0.163	0.926	±	0.026	0.289
Dlg1	0.648	±	0.068	0.010	0.072	±	0.017	0.013	0.079	±	0.006	0.744
Fzr1	0.181	±	0.057	0.177	0.067	±	0.016	0.195	0.074	±	0.018	0.792
Gpx3	0.011	±	0.002	0.017	0.776	±	0.101	0.017	0.855	±	0.111	0.627
Klf5	0.461	±	0.032	0.004	0.064	±	0.007	0.016	4.516	±	0.526	0.014
Kras	0.259	±	0.040	0.032	0.046	±	0.006	0.033	0.051	±	0.006	0.602
Nf1	0.473	±	0.035	0.001	1.719	±	0.079	0.001	1.894	±	0.087	0.210
Gsk3b	0.216	±	0.017	0.003	0.044	±	0.009	0.001	0.569	±	0.010	0.000
Pdgfb	0.921	±	0.082	0.009	0.060	±	0.002	0.237	1.056	±	0.027	0.001
Pik3r1	0.037	±	0.019	0.002	0.524	±	0.040	0.002	0.578	±	0.045	0.425
Prdm16	0.253	±	0.042	0.185	0.395	±	0.073	0.001	2.559	±	0.091	0.001
Prkaa2	0.123	±	0.022	0.033	0.007	±	0.001	0.036	0.268	±	0.036	0.019
Rap1gap	0.565	±	0.079	0.024	0.089	±	0.012	0.024	0.098	±	0.013	0.636
Robo1	0.354	±	0.113	0.322	0.207	±	0.026	0.382	0.229	±	0.029	0.617
sin3	0.060	±	0.007	0.011	0.576	±	0.056	0.011	0.634	±	0.062	0.519
**Striatum**												
Akt1	3.303	±	0.322	0.018	1.246	±	0.087	0.019	1.000	±	0.000	0.106
Akt2	0.168	±	0.010	0.173	0.237	±	0.034	0.145	0.868	±	0.300	0.169

Araf	1.261	±	0.219	0.046	5.464	±	0.994	0.170	0.807	±	0.143	0.040
Bcr	3.793	±	0.294	0.006	0.080	±	0.009	0.005	0.750	±	0.093	0.018
Bin1	2.073	±	0.351	0.046	0.585	±	0.077	0.697	2.375	±	0.615	0.098
Ccnd1	2.341	±	0.133	0.034	9.280	±	1.332	0.004	1.248	±	0.091	0.026
Crem	0.957	±	0.120	0.166	3.778	±	1.325	0.689	0.895	±	0.073	0.161
Dact1	0.502	±	0.008	0.033	2.225	±	0.324	0.002	1.035	±	0.029	0.066
Dlg1	1.742	±	0.186	0.890	1.796	±	0.312	0.012	0.088	±	0.007	0.032
Fzr1	0.340	±	0.036	0.026	2.399	±	0.349	0.008	0.083	±	0.020	0.022
Gpx3	1.442	±	0.146	0.019	2.979	±	0.290	0.065	0.955	±	0.124	0.010
Klf5	0.529	±	0.098	0.040	0.059	±	0.008	0.015	5.046	±	0.588	0.014
Kras	1.161	±	0.065	0.111	2.692	±	0.563	0.003	0.057	±	0.007	0.043
Nf1	2.639	±	0.247	0.060	7.526	±	1.308	0.157	2.116	±	0.097	0.053
Gsk3b	0.426	±	0.034	0.033	1.959	±	0.293	0.008	0.042	±	0.002	0.023
Pdgfb	1.011	±	0.072	0.675	0.898	±	0.227	0.009	0.295	±	0.008	0.117
Pik3r1	0.510	±	0.253	0.018	2.554	±	0.404	0.647	0.645	±	0.050	0.040
Prdm16	1.944	±	0.253	0.031	0.754	±	0.090	0.053	2.859	±	0.101	0.001
Prkaa2	0.774	±	0.578	0.443	0.226	±	0.024	0.723	0.537	±	0.090	0.066
Rap1gap	2.325	±	0.240	0.114	1.587	±	0.273	0.012	0.436	±	0.058	0.047
Robo1	3.544	±	0.475	0.032	1.539	±	0.277	0.020	0.255	±	0.032	0.042
sin3	0.180	±	0.014	0.011	1.780	±	0.173	0.014	0.709	±	0.069	0.015

Table S2. Pathways analysis for selected candidate genes

KEGG Pathway	N	P-Value	Genes involved
Glioma	15	5.05E-23	*BRAF, PDGFB, CDK6, AKT1, NRAS, IGF1R, CCND1, KRAS, ARAF, PDGFRA, MDM2, PDGFRB, PIK3CA, PIK3R1, AKT2*
Melanoma	15	2.45E-22	*BRAF, PDGFB, CDK6, AKT1, NRAS, IGF1R, CCND1, KRAS, ARAF, PDGFRA, MDM2, PDGFRB, PIK3CA, PIK3R1, AKT2*
Prostate cancer	15	3.63E-21	*BRAF, PDGFB, AKT1, NRAS, IGF1R, CCND1, KRAS, GSK3B, ARAF, PDGFRA, MDM2, PDGFRB, PIK3CA, PIK3R1, AKT2*
Chronic myeloid leukemia	12	3.60E-16	*AKT1, NRAS, CCND1, BCR, KRAS, BRAF, ARAF, PIK3CA, MDM2, CDK6, PIK3R1, AKT2*
Pathways in cancer	17	2.02E-14	*BCR, BRAF, PDGFB, CDK6, AKT1, NRAS, IGF1R, CCND1, KRAS, GSK3B, ARAF, PDGFRA, PIK3CA, MDM2, PDGFRB, PIK3R1, AKT2*
Endometrial cancer	10	5.95E-14	*AKT1, NRAS, CCND1, KRAS, BRAF, GSK3B, ARAF, PIK3CA, PIK3R1, AKT2*
Non-small cell lung cancer	10	1.47E-13	*AKT1, NRAS, CCND1, KRAS, BRAF, ARAF, PIK3CA, CDK6, PIK3R1, AKT2*
FoxO signaling pathway	12	3.15E-13	*AKT1, IGF1R, NRAS, CCND1, KRAS, BRAF, ARAF, PIK3CA, MDM2, PRKAA2, PIK3R1, AKT2*
PI3K-Akt signaling pathway	15	1.69E-12	*AKT1, IGF1R, NRAS, CCND1, KRAS, PDGFB, GSK3B, PDGFRA, PIK3CA, PDGFRB, MDM2, CDK6, PRKAA2, PIK3R1, AKT2*
Acute myeloid leukemia	9	1.09E-11	*AKT1, NRAS, CCND1, KRAS, BRAF, ARAF, PIK3CA, PIK3R1, AKT2*
HTLV-I infection	13	3.25E-11	*AKT1, NRAS, CCND1, KRAS, PDGFB, CREM, GSK3B, PDGFRA, PIK3CA, PDGFRB, PIK3R1, DLG1, AKT2*
Pancreatic cancer	9	3.78E-11	*AKT1, CCND1, KRAS, BRAF, ARAF, PIK3CA, CDK6, PIK3R1, AKT2*
Colorectal cancer	9	4.29E-11	*AKT1, CCND1, KRAS, BRAF, GSK3B, ARAF, PIK3CA, PIK3R1, AKT2*
Renal cell carcinoma	9	4.29E-11	*AKT1, NRAS, KRAS, BRAF, PDGFB, ARAF, PIK3CA, PIK3R1, AKT2*
Rap1 signaling pathway	12	6.43E-11	*AKT1, IGF1R, NRAS, KRAS, BRAF, RAP1GAP, PDGFB, PDGFRA, PIK3CA, PDGFRB, PIK3R1, AKT2*
ErbB signaling pathway	9	3.79E-10	*AKT1, NRAS, KRAS, BRAF, GSK3B, ARAF, PIK3CA, PIK3R1, AKT2*
Progesterone-mediated oocyte maturation	9	4.57E-10	*AKT1, IGF1R, FZR1, KRAS, BRAF, ARAF, PIK3CA, PIK3R1, AKT2*
Insulin signaling pathway	10	5.45E-10	*AKT1, NRAS, KRAS, BRAF, GSK3B, ARAF, PIK3CA, PRKAA2, PIK3R1, AKT2*
Proteoglycans in cancer	11	8.97E-10	*AKT1, IGF1R, NRAS, CCND1, KRAS, BRAF, ARAF, PIK3CA, MDM2, PIK3R1, AKT2*
Focal adhesion	11	1.14E-09	*AKT1, IGF1R, CCND1, BRAF, PDGFB, GSK3B, PDGFRA, PIK3CA, PDGFRB, PIK3R1, AKT2*
Choline metabolism in cancer	9	1.19E-09	*AKT1, NRAS, KRAS, PDGFB, PDGFRA, PIK3CA, PDGFRB, PIK3R1, AKT2*

Central carbon metabolism in cancer	8	1.39E-09	*AKT1, NRAS, KRAS, PDGFRA, PIK3CA, PDGFRB, PIK3R1, AKT2*
Thyroid hormone signaling pathway	9	3.44E-09	*AKT1, NRAS, CCND1, KRAS, GSK3B, PIK3CA, MDM2, PIK3R1, AKT2*
Ras signaling pathway	11	3.74E-09	*AKT1, IGF1R, NRAS, KRAS, PDGFB, NF1, PDGFRA, PIK3CA, PDGFRB, PIK3R1, AKT2*
Prolactin signaling pathway	8	4.94E-09	*AKT1, NRAS, CCND1, KRAS, GSK3B, PIK3CA, PIK3R1, AKT2*
Hepatitis C	9	1.26E-08	*AKT1, NRAS, KRAS, BRAF, GSK3B, ARAF, PIK3CA, PIK3R1, AKT2*
T cell receptor signaling pathway	8	5.81E-08	*AKT1, NRAS, KRAS, GSK3B, PIK3CA, PIK3R1, AKT2, DLG1*
B cell receptor signaling pathway	7	1.30E-07	*AKT1, NRAS, KRAS, GSK3B, PIK3CA, PIK3R1, AKT2*
Neurotrophin signaling pathway	8	1.94E-07	*AKT1, NRAS, KRAS, BRAF, GSK3B, PIK3CA, PIK3R1, AKT2*
Bladder cancer	6	2.39E-07	*NRAS, CCND1, KRAS, BRAF, ARAF, MDM2*
Signaling pathways regulating pluripotency of stem cells	8	4.02E-07	*AKT1, IGF1R, NRAS, KRAS, GSK3B, PIK3CA, PIK3R1, AKT2*
Regulation of actin cytoskeleton	9	4.75E-07	*NRAS, KRAS, BRAF, PDGFB, ARAF, PDGFRA, PIK3CA, PDGFRB, PIK3R1*
Hepatitis B	8	6.17E-07	*AKT1, NRAS, CCND1, KRAS, PIK3CA, CDK6, PIK3R1, AKT2*
mTOR signaling pathway	6	1.74E-06	*AKT1, BRAF, PIK3CA, PRKAA2, PIK3R1, AKT2*
VEGF signaling pathway	6	1.74E-06	*AKT1, NRAS, KRAS, PIK3CA, PIK3R1, AKT2*
MAPK signaling pathway	9	1.93E-06	*AKT1, NRAS, KRAS, BRAF, PDGFB, NF1, PDGFRA, PDGFRB, AKT2*
Chemokine signaling pathway	8	2.78E-06	*AKT1, NRAS, KRAS, BRAF, GSK3B, PIK3CA, PIK3R1, AKT2*
AMPK signaling pathway	7	3.20E-06	*AKT1, IGF1R, CCND1, PIK3CA, PRKAA2, PIK3R1, AKT2*
Fc epsilon RI signaling pathway	6	3.28E-06	*AKT1, NRAS, KRAS, PIK3CA, PIK3R1, AKT2*
Measles	7	7.84E-06	*AKT1, CCND1, GSK3B, PIK3CA, CDK6, PIK3R1, AKT2*
Small cell lung cancer	6	1.20E-05	*AKT1, CCND1, PIK3CA, CDK6, PIK3R1, AKT2*
Viral carcinogenesis	8	1.31E-05	*NRAS, CCND1, KRAS, PIK3CA, MDM2, CDK6, PIK3R1, DLG1*
MicroRNAs in cancer	8	1.92E-05	*NRAS, CCND1, KRAS, PDGFB, PDGFRA, PDGFRB, MDM2, CDK6*
Estrogen signaling pathway	6	2.14E-05	*AKT1, NRAS, KRAS, PIK3CA, PIK3R1, AKT2*
Cholinergic synapse	6	3.75E-05	*AKT1, NRAS, KRAS, PIK3CA, PIK3R1, AKT2*
Insulin resistance	6	3.91E-05	*AKT1, GSK3B, PIK3CA, PRKAA2, PIK3R1, AKT2*
Natural killer cell mediated	6	5.04E-05	*NRAS, KRAS, BRAF, ARAF, PIK3CA, PIK3R1*

cytotoxicity			
Long-term depression	5	5.16E-05	*IGF1R, NRAS, KRAS, BRAF, ARAF*
Sphingolipid signaling pathway	6	5.69E-05	*AKT1, NRAS, KRAS, PIK3CA, PIK3R1, AKT2*
Thyroid cancer	4	1.72E-04	*NRAS, CCND1, KRAS, BRAF*
Fc gamma R-mediated phagocytosis	5	2.16E-04	*AKT1, PIK3CA, BIN1, PIK3R1, AKT2*
Non-alcoholic fatty liver disease (NAFLD)	6	2.22E-04	*AKT1, GSK3B, PIK3CA, PRKAA2, PIK3R1, AKT2*
Gap junction	5	2.57E-04	*NRAS, KRAS, PDGFB, PDGFRA, PDGFRB*
HIF-1 signaling pathway	5	3.44E-04	*AKT1, IGF1R, PIK3CA, PIK3R1, AKT2*
Carbohydrate digestion and absorption	4	3.97E-04	*AKT1, PIK3CA, PIK3R1, AKT2*
Epstein-Barr virus infection	5	7.54E-04	*AKT1, PIK3CA, MDM2, PIK3R1, AKT2*
Axon guidance	5	9.36E-04	*NRAS, KRAS, ROBO1, GSK3B, SRGAP3*
Cell cycle	5	9.36E-04	*CCND1, FZR1, GSK3B, MDM2, CDK6*
Regulation of lipolysis in adipocytes	4	9.45E-04	*AKT1, PIK3CA, PIK3R1, AKT2*
Apoptosis	4	0.00134	*AKT1, PIK3CA, PIK3R1, AKT2*
Long-term potentiation	4	0.001537	*NRAS, KRAS, BRAF, ARAF*
Jak-STAT signaling pathway	5	0.001886	*AKT1, CCND1, PIK3CA, PIK3R1, AKT2*
Influenza A	5	0.003095	*AKT1, GSK3B, PIK3CA, PIK3R1, AKT2*
cAMP signaling pathway	5	0.005018	*AKT1, BRAF, PIK3CA, PIK3R1, AKT2*
Toll-like receptor signaling pathway	4	0.005999	*AKT1, PIK3CA, PIK3R1, AKT2*
TNF signaling pathway	4	0.006487	*AKT1, PIK3CA, PIK3R1, AKT2*
Chagas disease (American trypanosomiasis)	4	0.007532	*AKT1, PIK3CA, PIK3R1, AKT2*
Serotonergic synapse	4	0.007902	*NRAS, KRAS, BRAF, ARAF*
Aldosterone-regulated sodium reabsorption	3	0.008412	*KRAS, PIK3CA, PIK3R1*

Platelet activation	4	0.010126	*AKT1, PIK3CA, PIK3R1, AKT2*
Osteoclast differentiation	4	0.011713	*AKT1, PIK3CA, PIK3R1, AKT2*
Oxytocin signaling pathway	4	0.014484	*NRAS, CCND1, KRAS, PRKAA2*
Adipocytokine signaling pathway	3	0.026303	*AKT1, PRKAA2, AKT2*
p53 signaling pathway	3	0.026303	*CCND1, MDM2, CDK6*
Alcoholism	4	0.040302	*NRAS, KRAS, BRAF, ARAF*
Glucagon signaling pathway	3	0.044854	*AKT1, PRKAA2, AKT2*
Melanogenesis	3	0.04831	*NRAS, KRAS, GSK3B*
Endocytosis	4	0.05457	*IGF1R, PDGFRA, MDM2, BIN1*
Dopaminergic synapse	3	0.076022	*AKT1, GSK3B, AKT2*
Adrenergic signaling in cardiomyocytes	3	0.084393	*AKT1, CREM, AKT2*
Ubiquitin mediated proteolysis	3	0.084393	*FZR1, MDM2, PARK2*

Table S3. List of genes selected for phylogenetic and KEGG analysis

**Oncogenes**	**Tumor suppressor genes**
Abl1, Abl2, Akt1, Akt2, Birc3, Araf, Rhoa, Bax, Ccnd1, Bcl3, Bcl9, Bcr, Braf, Runx1, Cbl, Cdc25c, Cdh17, Klf6, Creb1, Csf1r, Csf3r, Cux1, Ddx6, Epha2, Elf4, Erbb2, Erbb3, Erbb4, Erg, Ewsr1, Fgfr1, Fgfr2, Fgfr4, Foxm1, Foxo1, Gata1, Gli1, Gli3, Gnai2, Gnaq, Grm1, Hlf, Hmga1, Idh1, Igf1r, Eif3e, Irf2, Irf4, Itk, Jak1, Jak2, Jun, Kit, Kras, Lck, Lmo2, Mcl1, Mdm2, Mef2c, Met, Mitf, Mllt1, Mpl, Myb, Mycn, Nedd4, Nfib, Nfkb2, Notch1, Npm1, Nras, Nup98, Pax2, Pdgfb, Pdgfra, Pdgfrb, Pik3ca, Pim1, Pik3r1, Plag1, Pml, Ppp2r1a, Ptpn11, Ralgds, Rara, Rel, Ros1, Set, Stil, Ski, Src, Ss18, Syk, Tal1, Tcf3, Tfe3, Tle1, Tmprss2, Tpr, Trio, Vav1, Zmym2, Pax8, Dek, Nup214, Ccdc6, Ncoa4, Yeats4, Hmga2, Ell, Ncoa3, Ppm1d, Fubp1, Ccnb2, Espl1, Akap9, Ikzf1,Tfg, Malt1, Fgfr1op, Psip1, Crtc1, Brd4, Suz12, Wwtr1, Foxp1, Eml4, Zbtb7a, Gopc, Prdm16, Aspscr1, Card11	*Per2, Erf, Ccdc136, Crem, Zfp36, Arntl, Cdkn1a, Dph1, Dcun1d3, Rasl10a, Rassf1, Nr4a1, Cxxc4, Npas2, Chd1, Dclre1a, Srgap3, Atf3, Smarca2, Ptch2, Notch2, Cldn1, Rap1gap, Lima1, Prdm5, Robo1, Plk2, Srpx, Klf5, Mapk10, Sox15, Marveld1, Bin1, Lsamp, Tspan32, Hltf, Bcr, Sall2, Prkaa2, Blnk, Gli1, Park2, Ptgdr, Rbbp7, Dact1, Cdk6, Fzr1, Cxcl14, Lrig3, Gpx3, Gsk3b, Hspd1, Nf1, Fbxw7, Samd9l, Dicer1, Map4k1, Bmpr1a, Dlg1, Nfatc2, Ppp2r5c, Llgl1, Ing4, Gnb2l1, Sfrp2, Dkk3, Hic1, Abi2*

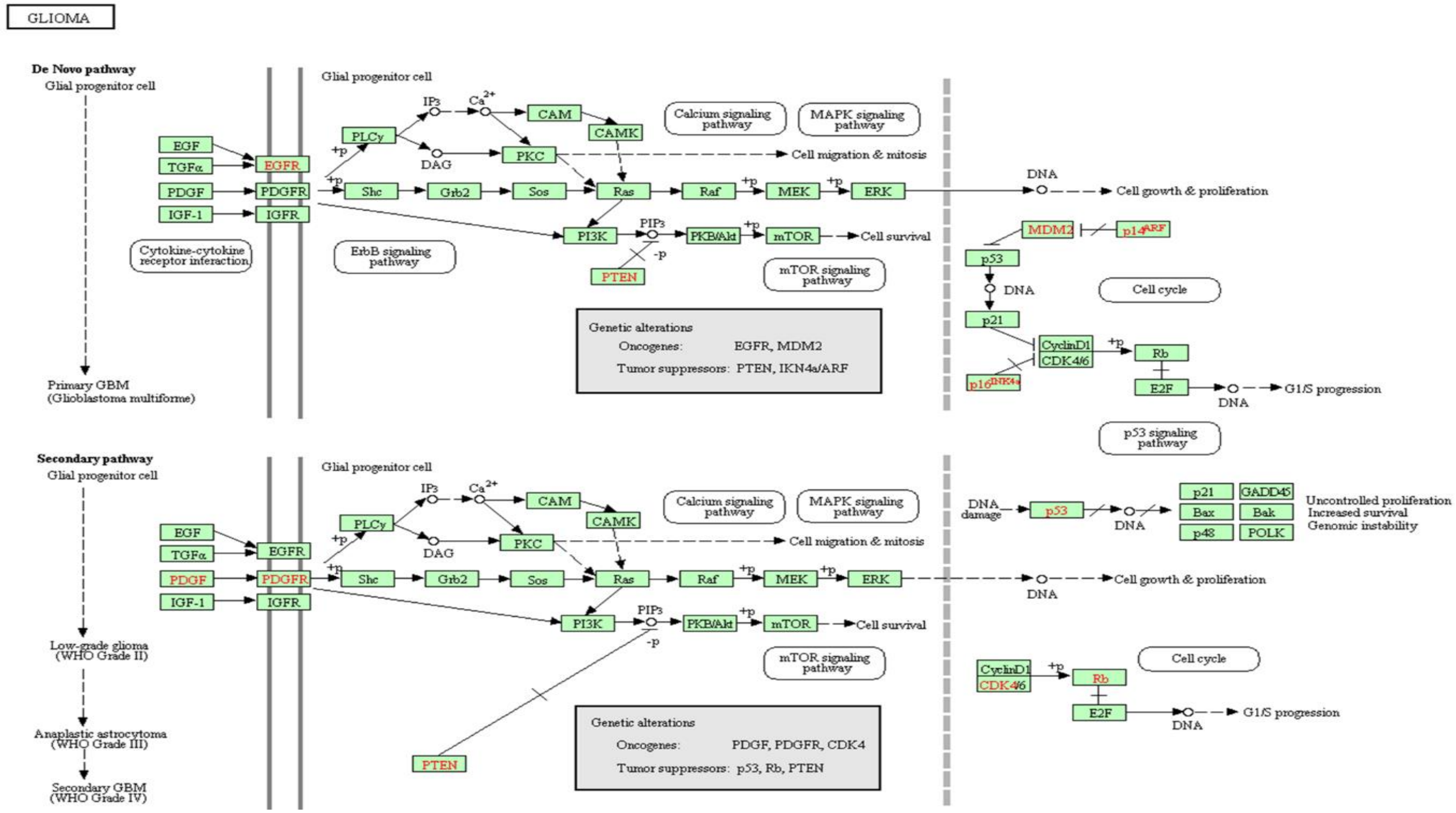

Figure S1. KEGG analysis of the selected gens revealed that a high percentage of the selected genes was associated with glioma pathway.

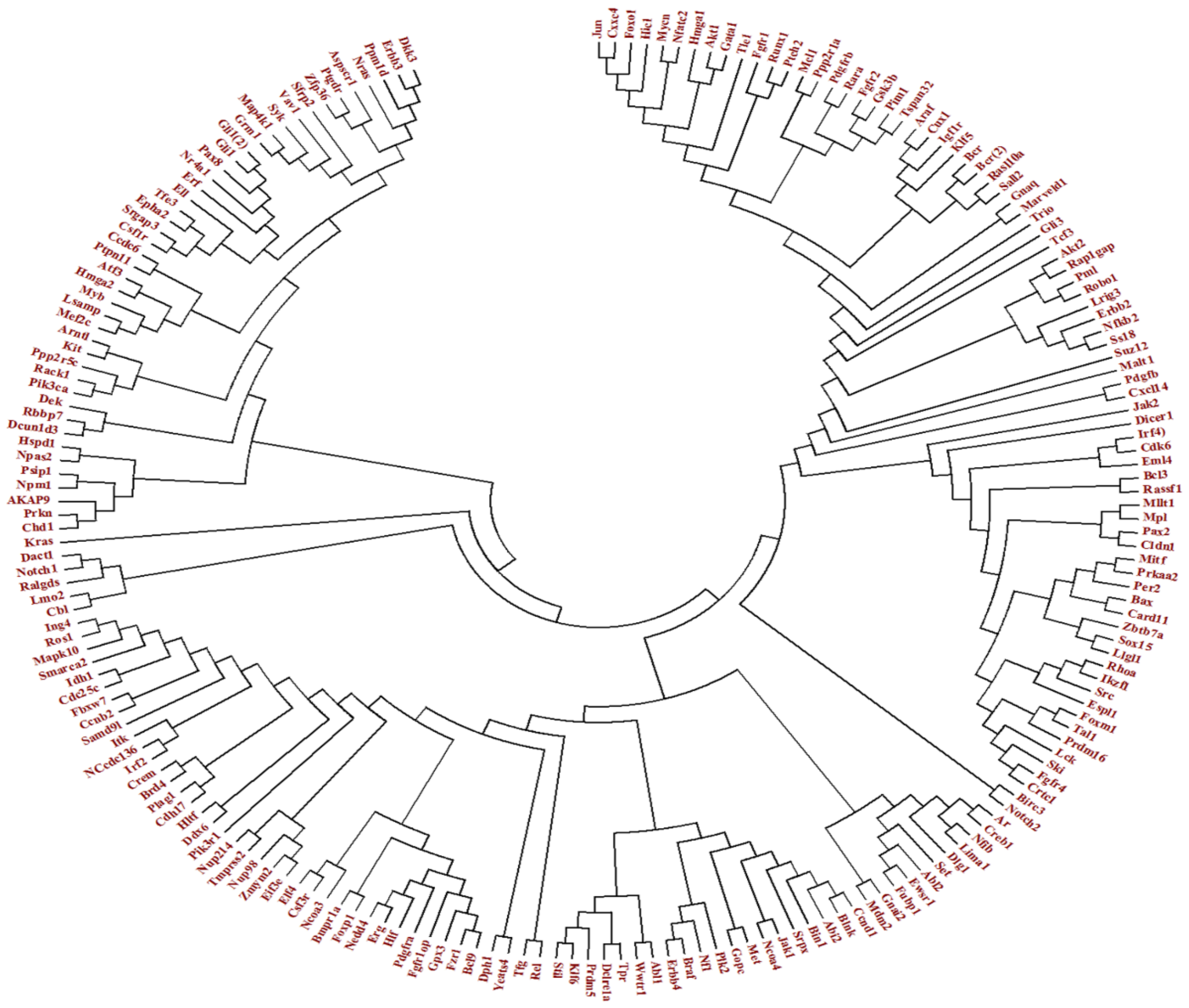

Figure S2. Phylogenetic tree for the selected 201 genes. These genes were found common in all the databases. The closely related genes were further analyzed.

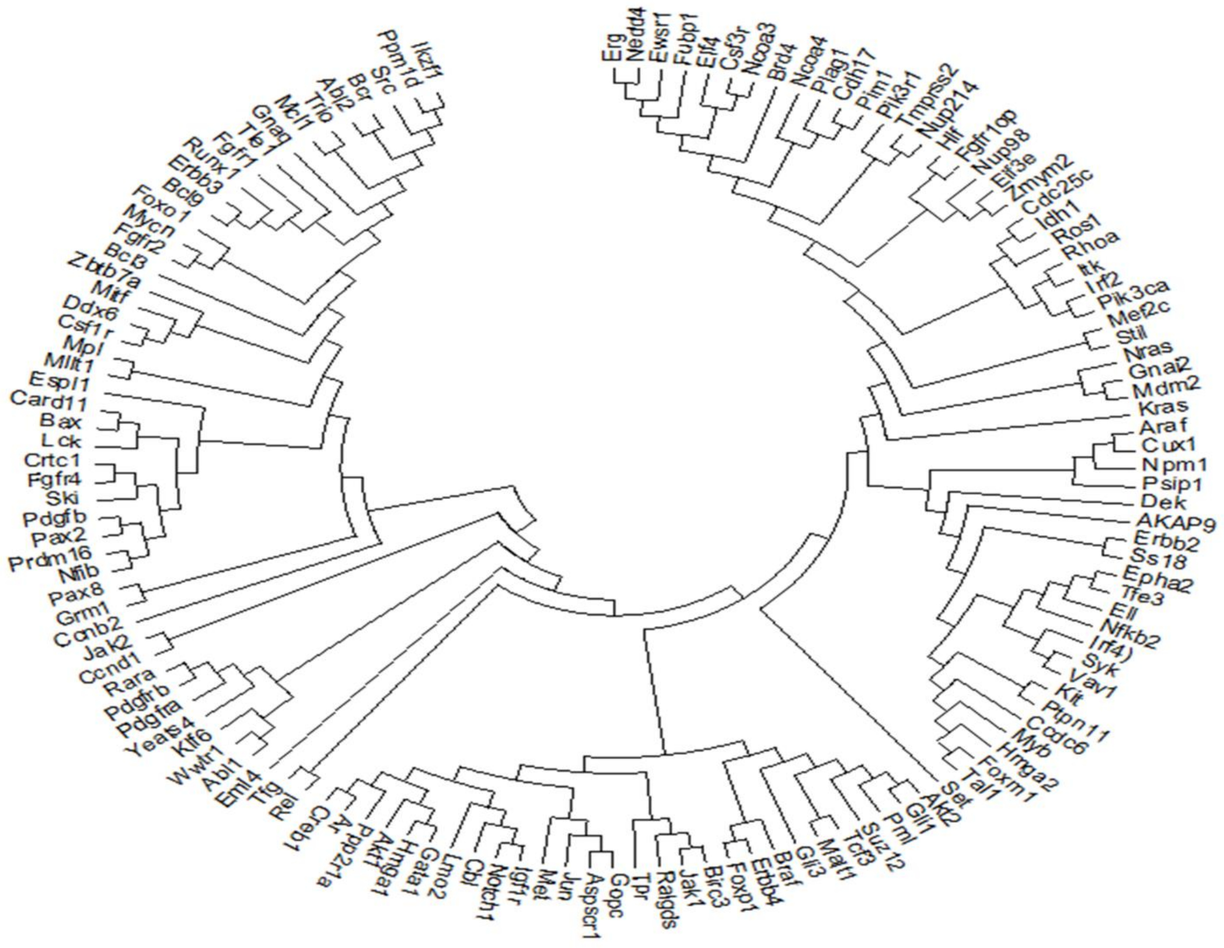

Figure S3. Phylogenetic analysis for the selected 133 oncogenes. These genes were found common in all the databases. The closely related genes were further analyzed.

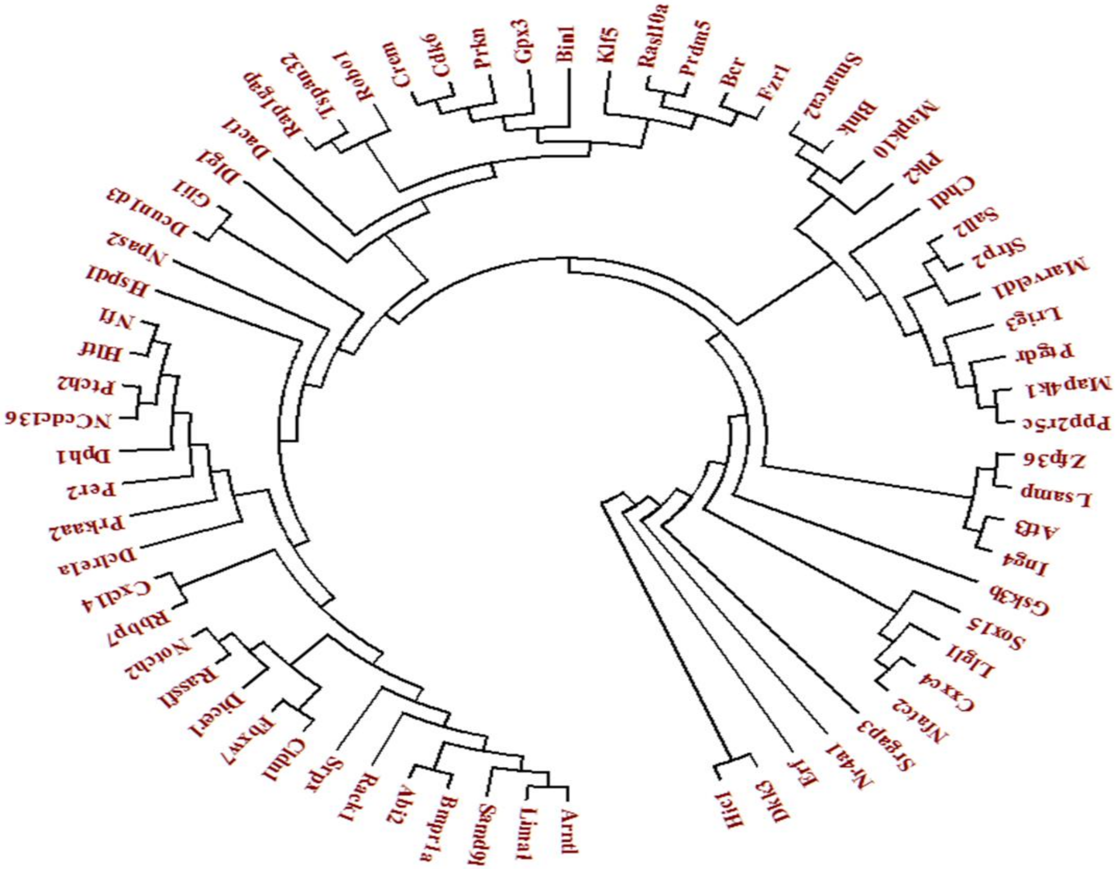

Figure S4. Phylogenetic tree for the selected 68 tumor suppressor genes. These genes were found common in all the databases. The closely related genes were further analyzed.
